# Crystal structures of human ENPP1 in apo and bound forms

**DOI:** 10.1107/S2059798320010505

**Published:** 2020-08-17

**Authors:** Matthew L. Dennis, Janet Newman, Olan Dolezal, Meghan Hattarki, Regina N. Surjadi, Stewart D. Nuttall, Tam Pham, Tom Nebl, Michelle Camerino, Poh Sim Khoo, Brendon J. Monahan, Thomas S. Peat

**Affiliations:** a Biomedical Manufacturing Program, CSIRO, 343 Royal Parade, Parkville, VIC 3052, Australia; b Cancer Therapeutics CRC, Parkville, VIC 3052, Australia; c Medicinal Chemistry Theme, Monash Institute of Pharmaceutical Sciences, Monash University, Parkville, VIC 3052, Australia; d Children’s Cancer Institute, Lowy Cancer Research Centre, UNSW, Sydney, NSW 2052, Australia; e Sir Peter MacCallum Department of Oncology, University of Melbourne, Melbourne, Victoria, Australia

**Keywords:** cancer, inhibitor structures, pyrophosphatase/phosphodiesterases, ENPP1

## Abstract

Cancer is one of the leading causes of mortality in humans, and recent work has focused on the area of immuno-oncology, in which the immune system is used to specifically target cancerous cells. Ectonucleotide pyrophosphatase/phosphodiesterase 1 (ENPP1) is an emerging therapeutic target in human cancers owing to its role in degrading cyclic GMP-AMP (cGAMP), an agonist of the stimulator of interferon genes (STING). The available structures of ENPP1 are of the mouse enzyme, and no structures are available with anything other than native nucleotides. Here, the first X-ray crystal structures of the human ENPP1 enzyme in an apo form, with bound nucleotides and with two known inhibitors are presented. The availability of these structures and a robust crystallization system will allow the development of structure-based drug-design campaigns against this attractive cancer therapeutic target.

## Abstract 2

Structures of the cancer target human ENPP1 have been determined in the apo form, bound to nucleotides and bound to known inhibitors of the enzyme.

## 1. Introduction   

Ectonucleotide pyrophosphatase/phosphodiesterase 1 (ENPP1), also known as PC-1, is found as a secreted protein in the extracellular matrix and as a membrane-bound protein intracellularly and on the cell surface. ENPP1 has a role in purin­ergic signalling through the hydrolysis of nucleotide triphosphates (NTPs) and cyclic nucleotides (cNMPs) to their corresponding nucleotide monophosphates (NMPs) (Stefan *et al.*, 2005 ▸; Namasivayam *et al.*, 2017 ▸). Apart from viral poxins (Eaglesham *et al.*, 2019 ▸), ENPP1 is the only known hydrolase of cyclic GMP-AMP (cGAMP), which is a second messenger generated by cyclic GMP-AMP synthase (cGAS) as an innate immune-system response to double-stranded DNA in the cytosol (Li *et al.*, 2014 ▸). In this way, cGAMP can signal viral infections as well as cancerous cells in proliferation. Although the charged nature of cGAMP is not conducive to passive diffusion across cell membranes, gap junctions permit intercellular transfer (Wu & Chen, 2014 ▸; Ablasser *et al.*, 2013 ▸) and transporters pump cGAMP both into and out of the cell (Carozza *et al.*, 2020 ▸; Ritchie *et al.*, 2019 ▸; Zhou *et al.*, 2020 ▸). cGAMP binds and activates the endoplasmic reticulum surface receptor stimulator of interferon genes (STING) to activate the production of type 1 interferons. Type 1 interferons are cytokines that trigger the innate and adaptive immune system used by the body to destroy virally infected or cancerous cells. In this context, STING activates the immune system to threats, whereas ENPP1 suppresses the immune response. Concordant with this immunosuppressive role, the expression of ENPP1 has been observed to correlate with the aggressiveness of astrocytic brain tumours (Aerts *et al.*, 2011 ▸) and to enhance bone metastasis from breast cancer (Lau *et al.*, 2013 ▸). ENPP1 thus represents an attractive target in cancer therapies.

In an effort to understand the molecular basis of this system, we have crystallized and determined the structures of human ENPP1 (hENPP1) bound to several compounds. We were able to crystallize the extracellular domains [residues 98–925, consisting of the somatomedin-B-like (SMB), catalytic and nuclease-like domains] that were expressed in mammalian cells, resulting in a protein with several post-translational modifications, which exceed those seen in the mouse ENPP1 (mENPP1) structures known to date. We have determined the apo structure as well as several structures co-crystallized with natural products, natural product analogues and small-molecule inhibitors. These compounds were further characterized using surface plasmon resonance (SPR) and differential scanning fluorometry (DSF) to determine their binding affinities to hENPP1. Additional analysis was performed by mass spectrometry to verify the post-translational modifications found in the crystal structures. To our knowledge, this is the first report of a fully human ENPP1 structure both with and without compounds bound.

## 2. Methods   

### 2.1. Cloning, expression and purification   

1 l of 293-F cells in 293 Freestyle medium supplemented with 5 µ*M* kifunensine was transfected with 1 mg hENPP1-HIS DNA using polyethylenimine at a DNA:PEI ratio of 1:3. The medium was harvested after seven days and filter-sterilized. The construct had a C-terminal 6×His tag and the protein was purified on a 5 ml affinity column (Ni Sepharose 6) at a flow rate of 10 ml min^−1^, washing with 15 column volumes of buffer 1 (290 m*M* NaCl, 3 m*M* KCl with 25 m*M* Tris pH 7.5) with 10 m*M* imidazole and then with another 15 column volumes of buffer 1 with 20 m*M* imidazole before elution with 250 m*M* imidazole in buffer 1. Gel filtration (16/60 Superdex 200 column) was run with a buffer consisting of 140 m*M* NaCl, 3 m*M* KCl, 25 m*M* Tris pH 7.5 supplemented with 50 µ*M* ZnCl_2_. Analysis of the gel-filtration data showed that the vast majority of the protein was monomeric, and the monomer peak was concentrated to 14.4 mg ml^−1^ and flash-frozen in aliquots, which were stored at −80°C. Approximately 7.5 mg of purified protein was obtained from the 1 l culture.

### 2.2. Crystallography   

The initial crystallization trials were set up at both 20 and 8°C in sitting drops consisting of 150 nl protein solution and 150 nl reservoir solution equilibrated against 50 µl reservoir solution in SD-2 plates (Molecular Dimensions, UK). Crystals first appeared as long rods in condition H2 [20%(*w*/*v*) polyethylene glycol (PEG) 3350, 0.2 *M* trilithium citrate] of the Shotgun screen (Fazio *et al.*, 2014 ▸) after five days only at 8°C. Using micro-seeding, crystal growth was reliably obtained overnight. The final refined range of crystallization conditions were protein at 7.5 mg ml^−1^ with 19–22%(*w*/*v*) PEG 4000, 240–270 m*M* trilithium, triammonium or tri­potassium citrate at 8°C. No crystals were obtained using protein purified from medium without kifunensine addition. The crystals initially diffracted to only moderate resolution (3–4 Å), but cryoprotecting them with diethylene glycol to 20%(*v*/*v*) and performing all crystal manipulations in a cold room allowed us to obtain data sets to beyond 3 Å resolution for each complex.

All data sets were obtained on the MX2 microfocus beamline at the Australian Synchrotron at −173°C. More than one 360° data set could often be obtained from a single crystal by moving down the crystal to expose fresh, non-irradiated material. The data were indexed using *XDS* (Kabsch, 2010 ▸) and scaled using *AIMLESS* (Evans & Murshudov, 2013 ▸). The initial (apo) structure was determined by molecular replacement with *Phaser* (McCoy *et al.*, 2007 ▸) using the structure of mENPP1 (PDB entry 4b56; Jansen *et al.*, 2012 ▸) as a model. The SMB domains were removed from PDB entry 4b56 and a second ensemble with these was run in *Phaser*. The space group was determined to be *P*2_1_2_1_2_1_, with two molecules in the asymmetric unit. Manual rebuilding was performed using *Coot* (Emsley *et al.*, 2010 ▸) and refinement used *REFMAC* (Winn *et al.*, 2011 ▸). Continuous density was seen for the *A* protomer for residues Val105–Phe921, whereas the *B* protomer had several small breaks in the density, particularly in the C-terminal region from residues 612 to 875. Subsequent structures were solved by using *Phaser* to perform molecular replacement with the apo structure as the model. Data-collection and refinement statistics are given in Table 1 ▸. The models and structure factors are available from the RCSB as PDB entries 6wet, 6weu, 6wev, 6wew and 6wfj.

### 2.3. Surface plasmon resonance (SPR)   

SPR experiments were performed using a Biacore T200 or S200 biosensor with a CM5 chip (GE Healthcare). hENPP1^98–925^ was coupled to the chip surface at 25°C in HBS-P+ running buffer [10 m*M* HEPES pH 7.4, 150 m*M* NaCl, 0.05%(*v*/*v*) Tween 20] after a 10 min activation with a 1:1 mixture of NHS/EDC [*N*-hydroxysuccinimide/*N*-ethyl-*N*′-(3-diethylaminopropyl)carbodiimide]. The protein was diluted to 25 µg ml^−1^ at pH 6.0 [10 m*M* 2-(*N*-morpholino)ethanesulfonic acid (MES), 0.005%(*v*/*v*) Tween 20] and injected to an immobilization level of 8–10 kRU. The surface was then blocked with 1 *M* ethanol­amine pH 8.0 for 7 min. SPR experiments were performed at 25°C in SPR running buffer consisting of 50 m*M* Tris pH 7.4, 150 m*M* NaCl, 50 µ*M* ZnCl_2_, 0.005%(*v*/*v*) Tween 20, 2%(*v*/*v*) DMSO. When performing dose–response testing, analytes were serially diluted (twofold or threefold), injected for 30–90 s contact time and allowed to dissociate back to baseline. The flow rate was 60 µl min^−1^. Binding sensorgrams were processed, solvent-corrected and double-referenced using the *Scrubber* software (BioLogic Software, Australia). Responses at equilibrium for each analyte were fitted to a 1:1 steady-state affinity model that was available within *Scrubber*. Sensorgrams and binding isotherms are displayed in Supplementary Fig. S5.

### 2.4. Differential scanning fluorimetry (DSF)   

DSF experiments were performed using a Bio-Rad CFX96 thermocycler, with the fluorescence intensity measured with excitation and emission at 490 and 570 nm, respectively. Plates were heated from 20 to 100°C at a heating rate of 1.0°C min^−1^. Protein was present at a final concentration of 15 µg ml^−1^. The well volume was 20 µl. The buffer was 50 m*M* Tris–HCl pH 7.4, 150 m*M* NaCl, 50 µ*M* ZnCl_2_. Data were acquired using Bio-Rad *CFX Manager* (version 3.1) and were processed using *Meltdown* (Rosa *et al.*, 2015 ▸), with *T*_m_ values calculated from the negative peak of the first derivative of the melting curve. Δ*T*_m_ values were obtained by subtracting the *T*_m_ of the ligand-bound enzyme from that of the apoenzyme [0 or 1%(*v*/*v*) DMSO present as a control, depending on the stock solvent]. Ligands and controls were present in triplicate. Melting curves are displayed in Supplementary Fig. S6.

### 2.5. Mass spectrometry   

Intact mass determination of kifunensine-treated, affinity-purified hENPP1-HIS samples was achieved by LC-MS as described previously (Newman *et al.*, 2019 ▸). N-linked glycoprofiling was achieved by hydrophilic liquid chromatography with fluorescence and mass-spectrometry readouts (HILIC-FL-MS). PNGase F-released glycan samples were prepared using the GlycoWorks RapiFluor-MS N-glycan kit according to the manufacturer’s protocol (Waters Corporation, USA). RapiFluor-MS-labelled N-glycans were separated on an AdvanceBio Glycan Mapping column (Agilent Technologies; 120 Å, 2.1 × 250 mm, 2.7 µm) using an UltiMate 3000 HPLC system (Thermo Fisher, USA) connected to a MicrOTOF-Q II mass spectrometer (Bruker-Daltonics, Germany). The HILIC-FL-MS data were analysed using the *Byomap* glycan-mapping software (Protein Metrics, USA).

For peptide sequencing, hENPP1 samples were digested with trypsin using the SP3 digestion protocol as described in Hughes *et al.* (2014 ▸). Tryptic peptides were separated using a 60 min linear gradient of 3–40%(*v*/*v*) acetonitrile in 0.1%(*v*/*v*) formic acid on an UltiMate nanoUPLC system (Thermo Fisher, USA) equipped with an Acclaim PepMap nano-trap column (Dionex C18, 100 Å, 75 µm × 2 cm) and an Acclaim PepMap RSLC analytical column (Dionex C18, 100 Å, 75 µm × 50 cm). Tandem mass spectrometry (MS/MS) data were obtained in positive-ionization mode on an Orbitrap Fusion Lumos Mass Spectrometer (Thermo Fisher, USA). The mass spectrometer was operated in data-dependent acquisition mode, whereby full MS1 spectra were acquired in positive mode at a resolution of 120 000 and the top 20 most intense peptide ions with charge states of 2–5 were fragmented using high-energy collision (HCD), and MS2 spectra were acquired in the Orbitrap at a resolution of 15 000.

High-resolution MS/MS data were searched against a focused decoy database containing hENPP1-HIS, *Escherichia coli* and common contaminant protein sequences using the *Byonic* search engine (Protein Metrics, USA) with a tolerance of 5 p.p.m. for precursor ions and 20 p.p.m. for fragment ions. Enzyme specificity was tryptic and allowed up to two missed cleavages per peptide. Variable modifications were set for NH_2_-terminal acetylation or protein N-termini, oxidation of methionine or tryptophan, dioxidation of tryptophan, deamidation of asparagine or glutamine and phosphorylation of serine, threonine or histidine. *Byonic* glycopeptide searches against a human N-linked glycan database facilitated confident peptide identifications (FDR < 1%) with relative quantification of post-translationally modified hENPP1 peptides based on spectrum counting.

### 2.6. Chemical synthesis   

ADU-S100 was purchased from MedChemExpress. QPS2 was synthesized as described previously (Patel *et al.*, 2009 ▸). Ex54 synthesis was based on the methods found in Gallatin *et al.* (2019 ▸) (Example 54).

## 3. Results and discussion   

**Figure 1:**
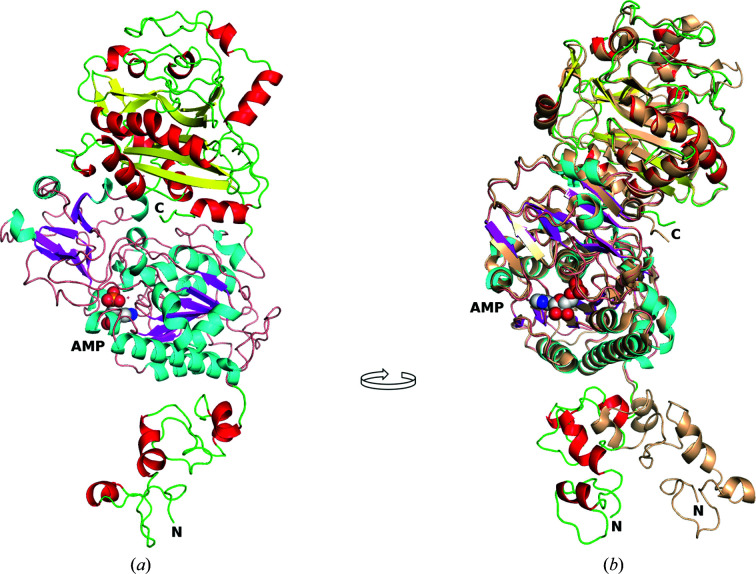
Structure of hENPP1 with AMP and comparison with mouse ENPP1 (PDB entry 4b56). (*a*) The SMB domain is at the N-terminus and is shown with red helices, the catalytic domain is shown with cyan helices and magenta β-strands with AMP represented as spheres, and the nuclease-like domain is at the top of the figure and is shown with red helices and yellow β-strands. (*b*) The image is rotated approximately 90° clockwise and mouse ENPP1 (PDB entry 4b56) is superposed on the human structure. The mouse structure is coloured monochrome wheat with the N- and C-termini labelled. This figure was generated using *PyMOL* and *GIMP*.

Overall, the human ENPP1 structures presented are similar to those of the secreted mouse ENPP1 (see Fig. 1 ▸; Kato *et al.*, 2012 ▸, 2018 ▸; Jansen *et al.*, 2012 ▸). A high sequence identity (79.7%), particularly for the catalytic domain (88.4%), allowed the structure of the mouse enzyme, PDB entry 4b56, to be used to obtain the phases *via* molecular replacement. The catalytic and nuclease-like domains of hENPP1 align well with those of mENPP1, with an r.m.s.d. of less than 1 Å (0.87 Å) between the C^α^ atoms of the two structures (with 706 of the 817 residues of the *A* protomers aligned using the *SSM* algorithm in *Coot*). The SMB domains, in contrast, show a divergent orientation between species relative to the catalytic domain (Fig. 1 ▸ *b*). Alignment of the SMB domains specifically (residues 105–187 of hENPP1 and 88–168 of mENPP1) also gives a C^α^-atom r.m.s.d. of <1 Å. Molecular replacement was thus performed by separating the SMB domains from the catalytic and nuclease-like domains and running independent searches. The membrane-bound hENPP1 has been shown to be a disulfide-linked dimer (Dimatteo *et al.*, 2013 ▸; Gijsbers *et al.*, 2003 ▸). The N-terminus of the protein makes the majority of the crystal contacts, with the C-terminal nuclease-like domain having few contacts in the *A* protomer (Ser627, Asn623, Lys725, Ser727, Phe728, Lys730, Arg821, Asn822 and the glycosylated Asn731 with a range of 3.4–4.5 Å) and none in the *B* protomer (>5 Å) (see Supplementary Fig. S1). As the hENPP1 construct is based on the monomeric, secreted form of the protein and the disulfide bond forming the dimer is found in the intramembrane region (which is missing in our construct), and the buried surface area between the two protomers is only 740 Å^2^, we conclude that the two protomers observed in the asymmetric unit are the result of crystal packing and are not a biologically relevant dimer (Krissinel & Henrick, 2007 ▸; Jansen *et al.*, 2012 ▸).

Fully glycosylated hENPP1 did not produce crystals. The cells were treated with kifunensine during protein production, which reduced the heterogeneity of glycosylation on the protein (see the mass-spectrometric data; Supplementary Fig. S2). Although the overall mass of glycosylation (about 14.5 kDa) was about the same in both the kifunensine-treated and the untreated cells, treatment reduced the heterogeneity significantly and resulted in crystals that diffracted to beyond 3 Å resolution. The hENPP1 protein has nine listed potential glycosylation sites in UniProt (ID P22413) and we see electron density for glycosylation at many of these (Asn285, Asn341, Asn477, Asn585 and Asn731), with two potential sites (Asn700 and Asn748) being in regions that have minimal density, leading to uncertainty regarding the glycosylation at these sites. In areas of well defined density, Asn179 and Asn643 do not have any extra density that would suggest glycosylation. Mass-spectrometric analysis shows evidence for glycosylation at all nine of the potential sites for hENPP1, although the level of glycosylation varies from 10 to 99%, with Asn179 and Asn748 having little glycosylation (10–27%), Asn285 being almost fully modified and the rest having between 36% and 100% glycosylation (see Supplementary Fig. S2). The glycosylation profiles seen in the crystallographic and mass-spectrometric results align reasonably well and demonstrate heterogeneity in the glycan composition and structure of hENPP1.

One unusual feature that is observed in some of the structures is a phosphorylated histidine residue at position 486. Mass-spectrometric analysis did not detect phosphorylation of His486, but was able to unambiguously demonstrate phosphorylation of His873 (see Supplementary Figs. S2 and S3) and Thr256. Unfortunately, the area around His873 is disordered in both the *A* and the *B* protomers. In addition, two different batches of protein were used to set up crystallization experiments, and one of these resulted in clear density (>7σ difference density peak) for the His486 phosphate group while the other did not. As the cells and protein were treated similarly in each case (the one known difference is the addition of 50 µ*M* ZnCl_2_ in the gel-filtration step), it is unclear what led to this phosphorylation at His486 in only one of the samples. Additional zinc was added to the gel-filtration protocol as we found that one of the two Zn atoms in the active site was either missing or not fully occupied. The apo structure has only a single Zn atom in both the *A* and *B* protomers. The PDB entry 6wev and 6wew structures (the complexes with QPS2 and Ex54, respectively) both have one site with two Zn atoms and one site with just a single Zn atom. Thr256 is more consistently phosphorylated, but the phosphate seems to be displaced by many of the compounds added to the crystals. Overall, the data are consistent with variable phosphorylation of histidine residues His486 and His873 of hENNP1. The role that phosphorylation of histidines might have on the biological function of hENPP1 is unclear.

**Figure 2:**
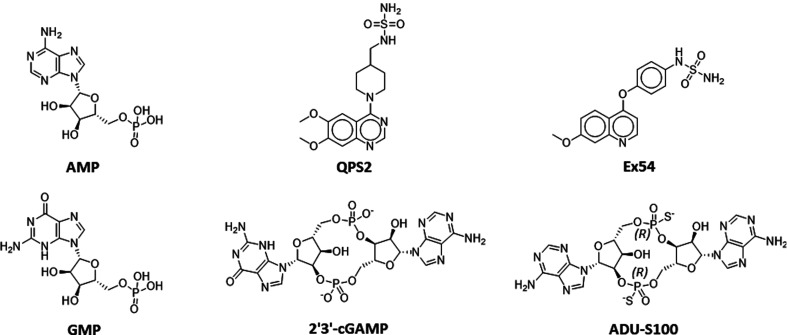
Chemical structures of the compounds. The structures of AMP, GMP and cGAMP and the synthesized compounds ADU-S100, QPS2 and Ex54 are shown.

The apo and compound-bound hENPP1 structures (the structures of the compounds are shown in Fig. 2 ▸) do not have any major shifts in domain positions, and changes between the structures are limited to a few residues in the active site (Figs. 1 ▸ and 3 ▸). Two turns present Asn277 and Leu290 towards the active site. Asn277 adopts two distinct conformations, whereas Leu290 has a single conformer that moves slightly to accommodate bound compounds. Ser377 is the only other active-site residue that is observed to adopt distinct conformations in the hENPP1 structures.

AMP is the product of the hydrolysis of ATP or cGAMP by ENPP1, and we found good density for AMP when it was co-crystallized with hENPP1 (Fig. 4 ▸ *a*). It is bound in a very similar fashion to that in the mENPP1–AMP structure (PDB entry 4gtw; Kato *et al.*, 2012 ▸), with the nucleobase stacked between Tyr340 and Phe257 and the N1 atom hydrogen-bonding to Lys295 (2.9–3.1 Å). The phosphate forms a complex network of interactions with the zinc ions in the active site, in addition to Asp218, Asn277, Thr256, Asn376, His380, His424 and His535. There is a small difference in the orientation of the ribose ring between the two structures and a hydrogen bond to Tyr340 is absent in hENPP1; however, this does not affect the orientation of the rest of the molecule.

**Figure 3:**
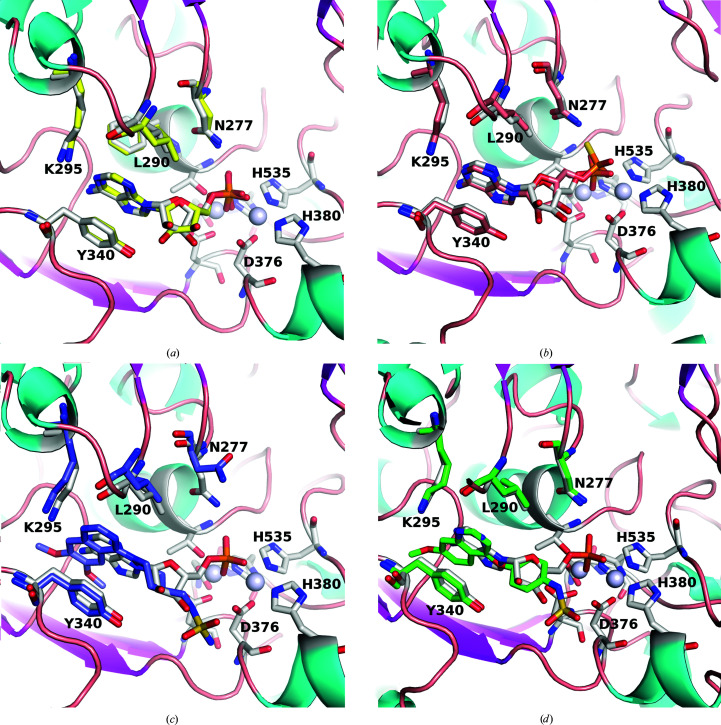
Relationship of structures with bound AMP. (*a*) The human and mouse (PDB entry 4gtw) structures of ENPP1 bound to AMP. hENPP1 has grey C atoms and mENPP1 has yellow C atoms for both the AMP molecules and the surrounding residues highlighted in stick representation (Asn277, Leu290, Lys295 and Trp340). The two Zn atoms are shown as grey spheres and the active-site residues are also highlighted in stick representation (three of these, Asp376, His380 and His535, are labelled). (*b*) hENPP1 with AMP superposed with the hydrolysed product of ADU-S100, with hENPP1 with AMP using the same colour scheme as in (*a*) and ADU-S100 coloured burnt orange. (*c*) hENPP1 with AMP superposed with hENPP1 with QPS2 (purple C atoms). (*d*) hENPP1 with AMP superposed hENPP1 with Ex54 (green C atoms). This figure was generated using *PyMOL* and *GIMP*.

**Figure 4:**
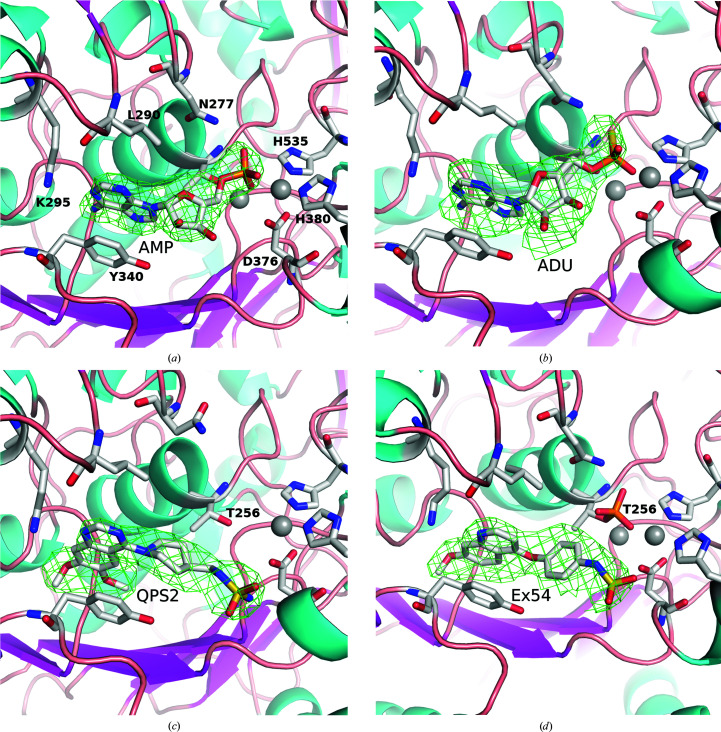
OMIT maps of compounds bound to hENPP1. 3σ difference OMIT maps of AMP (*a*), ADU-S100 (*b*), QPS2 (*c*) and Ex54 (*d*). The orientation is approximately the same as that in Fig. 3 ▸, with the Zn atoms shown as grey spheres and various residues in the binding site highlighted in stick representation. Note that Ex54 is bound such that Thr256 is still phosphorylated. This figure was generated with *PyMOL* and *GIMP*.

ADU-S100 is a cyclic dinucleotide composed of two AMP molecules linked by phosphorothioate moieties (*R*_p_,*R*_p_) in a 2′–5′ and 3′–5′ configuration (‘mixed linkage’; Corrales *et al.*, 2015 ▸). This compound is a STING agonist designed as an analogue of cGAMP, with the aim of improving affinity as well as reducing degradation by phosphodiesterases. The observed ligand density suggests that the compound ADU-S100 was hydrolysed during the crystallization experiment, leaving a thiophosphate equivalent of an AMP molecule bound in the active site (Figs. 3 ▸ *b* and 4 ▸ *b*). The adenine ring is essentially in the same position as found in the AMP-bound structure (0.4–0.5 Å movement in the *A* protomer; a slight twist in the *B* protomer leads to a maximum difference of 1 Å). The thiophosphate moiety has the S atom oriented towards Asn277 (2.2 Å distance to the side chain for the *A* protomer; the *B* protomer has Asn277 in a different rotamer position), with the thiophosphate further from the active-site Zn ions than the analogous phosphate of native mononucleotides. This movement is likely to result from the larger S atom being unable to maintain the close distance to Thr256 (2.6 Å) observed in the AMP-bound structure. The dictionary (cif) file initially had the sulfur–phosphate bond default distance set to 2.15 Å, but this was modified to 1.95 Å to correspond to the literature, which estimates this bond to be between a single and double bond, effectively with a bond order of 1.5 (Frey & Sammons, 1985 ▸).

The ENPP1 inhibitors Ex54 (Gallatin *et al.*, 2019 ▸) and QPS2 (Shayhidin *et al.*, 2015 ▸) have a modified quinoline or quinazoline core (monomethoxylated or dimethoxylated, respectively) that packs between Phe257 and Tyr340 and forms a hydrogen bond to Lys295 (2.8–3.2 Å), analogous to the adenine in the AMP-bound structure (Figs. 3 ▸ *c*, 3 ▸ *d*, 4 ▸ *c*, 4 ▸ *d* and 5 ▸). The quin(az)oline projects deeper into the pocket than the adenine ring, making additional interactions with Phe321, Pro323, Asp326 and Tyr371. In the AMP-bound structure, two water molecules are hydrogen-bonded to the N6 position. These are displaced in the QPS2-bound and Ex54-bound structures, and a hydrogen bond between the 7-methoxy group and Trp322 is introduced (3.0–3.2 Å). The methoxy moiety also appears in close contact with the carbonyl of Trp322 (2.4–2.8 Å), which may offset the affinity gains from the introduced hydrogen bond. Ex54 and QPS2 extend out of the adenine pocket via benzene and piperidine rings, respectively, which occupy a similar space to that of the ribose ring of AMP. The sulfamide groups of QPS2/Ex54 do not project towards the zinc site (as per the phosphate of AMP), but instead form hydrogen bonds to Glu373, Asp376 and Ser377. The sulfamide of Ex54 is flipped in the *B* protomer, projecting towards the bound phosphate, and therefore does not make the above inter­actions (Supplementary Fig. S4). This may suggest that the sulfamide moiety does not make particularly strong or specific interactions with the protein; however, the potency of QPS2 and related analogues has been shown to vary significantly if the linker length to the sulfamide is altered (Patel *et al.*, 2009 ▸).

Surface plasmon resonance experiments on a set of compounds reveal several interesting features (Table 2 ▸ and Supplementary Fig. S5). The adenine nucleotides (ADP and AMP; *K*_d_ = 50 and 103 n*M*, respectively) bind more tightly to hENPP1 than other native nucleotides, concordant with the preference for ATP observed previously in kinetic analyses (Kato *et al.*, 2012 ▸; Namasivayam *et al.*, 2017 ▸). GMP displays slightly weaker binding (*K*_d_ = 470 n*M*), while the pyrimidine nucleobases show a more marked loss of affinity (CMP, TMP and UMP all have a *K*_d_ of >1 µ*M*). The cyclic dinucleotide compounds tested also display a loss of affinity relative to AMP (3′3′-cGAMP and ADU-S100 have a *K*_d_ of 1.3 µ*M* and 660 n*M*, respectively). Previous research suggests that 3′3′-cGAMP and phosphothioate cyclic dinucleotides would be resistant to hydrolysis on the short timescale of an SPR experiment (60 s; Li *et al.*, 2014 ▸).

Ex54 and QPS2 (*K*_d_ = 100 and 55 n*M*, respectively) display similar affinities to that of ATP. All of the drug-like compounds would have difficulty competing with AMP/ADP/ATP in the cell as the intracellular concentrations of the adenosine compounds are quite high (millimolar). The concentrations of adenine nucleotides found extracellularly are significantly lower (in the low-nanomolar range), so competition with the adenosine nucleotides should be less of an issue extracellularly (Traut, 1994 ▸; Di Virgilio & Adinolfi, 2017 ▸). It has been reported that hENPP1 regulates the extracellular concentration of 2′3′-cGAMP but not the intracellular concentration of this second messenger, so inhibitors of the extracellular hENPP1 should have less competition (Carozza *et al.*, 2020 ▸).

Additional qualitative binding studies were performed using DSF (see Supplementary Fig. S6). These data showed that the compounds tested increased the thermal melting temperature by about 2–7°C. Although AMP showed the largest Δ*T*_m_ of the NMPs, there was otherwise little notable correlation between the DSF melting temperature and the measured SPR affinity.

## 4. Conclusions   

ADU-S100 is a STING agonist that is currently in clinical trials (Meric-Bernstam *et al.*, 2019 ▸), while QPS2 and Ex54 are published ENPP1 inhibitors. These compounds were tested in order to understand how they bind and inhibit hENPP1. The structures presented elucidate the binding modes of these compounds and how they differ from nucleotide binding. The fused rings of QPS2 and Ex54 occupy the same region as the adenine of AMP but extend further into the pocket, displacing the waters that hydrogen-bond to N6 of AMP. The sulfamide moieties of these inhibitors do not seem to make strong interactions in the catalytic site, unlike the phosphate of AMP. As ADU-S100 was hydrolysed during the experiment, one can only speculate as to the additional interactions that the full (nonhydrolysed) molecule might make with the binding site of hENPP1. Species differences between the human and mouse structures are additionally clarified. We were able to obtain high multiplicity and completeness for the crystallo­graphic data, and the density for both the compounds and the catalytic site was quite well defined, particularly for these moderate-resolution data (2.5–2.9 Å resolution). The crystallographic results are fairly clear: there are no major conformational changes upon the binding of these compounds, and there are no major differences in nucleotide binding between the mouse and human structures. The SPR data show more disparity between the compounds, with values starting about 50 n*M* and increasing to 600 n*M* for the synthesized compounds and from 50 n*M* to 1.8 µ*M* for compounds found naturally in the body. The naturally occurring adenosine compounds bind quite tightly, which could pose challenges for intracellular inhibition; competitive inhibitors of hENPP1 may thus rely on the low concentration of extracellular nucleotides for efficacy.

## Supplementary Material

PDB reference: human ENPP1, apo, 6wet

PDB reference: complex with ADU-S100, 6weu

PDB reference: complex with QPS2, 6wev

PDB reference: complex with Ex54, 6wew

PDB reference: AMP-bound, 6wfj

Supplementary Figures. DOI: 10.1107/S2059798320010505/cb5120sup1.pdf

## Figures and Tables

**Figure 5:**
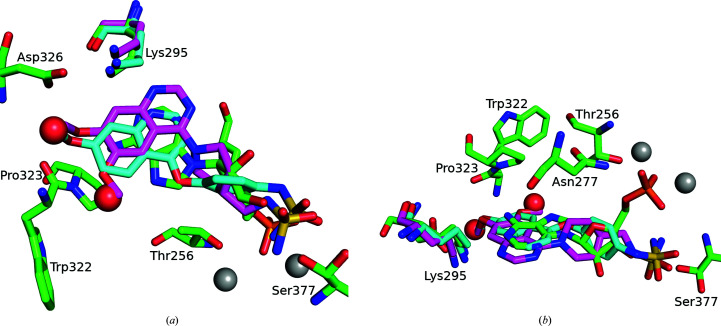
Superposition of Ex54 and QPS2 on AMP in hENPP1. AMP has green C atoms, Ex54 has cyan C atons and QPS2 has magenta C atoms. The Zn atoms of the active site are shown as grey spheres and the two water molecules found in the AMP-bound structure but not in the inhibitor-bound structures are shown as red spheres. The methoxy moieties of the inhibitors occupy approximately the same space as that occupied by the two waters in the AMP-bound structure. (*a*) and (*b*) show two different orientations to highlight that the fused rings are all in the same plane and that the phosphate moiety of AMP is found in a different orientation to that of the sulfamides of the two inhibitors.

**Table 1:** Data-collection and refinement statistics Values in parentheses are for the highest resolution bin.

PDB code	6wet	6wfj	6weu	6wev	6wew
Compound	Apo	AMP	ADU-S100	QPS2	Ex54
Data collection					
Space group	*P*2_1_2_1_2_1_	*P*2_1_2_1_2_1_	*P*2_1_2_1_2_1_	*P*2_1_2_1_2_1_	*P*2_1_2_1_2_1_
*a*, *b*, *c* (Å)	83.1, 158.9, 209.8	83.0, 159.7, 209.3	83.0, 160.1, 209.6	82.3, 159.0, 209.2	83.1, 158.9, 209.8
α, β, γ (°)	90, 90, 90	90, 90, 90	90, 90, 90	90, 90, 90	90, 90, 90
Resolution (Å	47.3–2.60 (2.65–2.60)	47.4–2.50 (2.54–2.50)	47.6–2.65 (2.70–2.65)	47.3–2.90 (2.98–2.90)	47.9–2.73 (2.79–2.73)
*R*_merge_	0.270 (3.380)	0.208 (2.032)	0.284 (2.937)	0.430 (3.786)	0.348 (2.489)
*R*_p.i.m._	0.053 (0.916)	0.041 (0.567)	0.055 (0.816)	0.085 (0.740)	0.105 (0.765)
CC_1/2_	0.998 (0.539)	0.999 (0.650)	0.997 (0.508)	0.993 (0.569)	0.990 (0.548)
〈*I*/σ(*I*)〉	12.9 (1.0)	15.1 (1.3)	12.1 (1.0)	9.9 (1.1)	8.4 (1.3)
Completeness (%)	100 (100)	99.9 (99.5)	100 (100)	99.9 (99.7)	100 (100)
Multiplicity	26.1 (14.5)	26.1 (13.7)	26.9 (13.7)	26.0 (26.6)	11.8 (11.6)
Refinement					
Resolution (Å)	44.4–2.60	47.4–2.50	47.6–2.65	47.3–2.90	47.9–2.73
Unique reflections	81708	91848	77722	58507	72171
*R*_work_/*R*_free _(%)	21.0/24.4	19.9/22.2	20.7/23.9	21.1/25.3	21.0/24.1
No. of atoms					
Total	12979	13320	13105	13052	13250
Protein	12875	13044	12939	12967	12992
Metal (Zn)	2	4	4	3	3
Ligand	0	46	46	52	48
Water	72	214	122	20	185
*B* factors (Å^2^)					
Overall	68.7	60.3	60.1	62.6	51.5
Protein (protomer *A*/*B*)	66.6/87.0	58.1/76.2	57.5/77.3	59.4/79.8	47.3/68.3
Zn (1st/2nd)	60.3/—	40.1/41.3	51.3/80.0	53.8/103.9	49.0/79.3
Ligand (protomer *A*/*B*)	—	53.3/62.4	88.5/84.1	45.6/65.8	60.3/75.5
Water	49.1	45.0	40.8	32.3	31.6
R.m.s. deviations					
Bond lengths (Å)	0.005	0.005	0.005	0.006	0.005
Bond angles (°)	1.362	1.373	1.356	1.458	1.393

**Table 2:** Binding of compounds to hENPP1

Compound	*K*_d_ † (n*M*)	Δ*T*_m_ ‡
AMP	103 ± 8	+2.7
ADP	56 ± 3	+3.3
GMP	470 ± 60	+1.0
UMP	1100 ± 300	+1.8
CMP	1500 ± 300	+2.1
TMP	1800 ± 300	+1.2
3′3′-cGAMP	1300 ± 300	+1.2
QPS2	55 ± 6	+4.5
Ex54	100 ± 20	+3.8
ADU-S100	660 ± 50	+7.0

† Determined by SPR, triplicates ± SD.

‡ DSF testing with compounds at 100 µ*M*, apart from 3′3′-cGAMP, which was tested at 10 µ*M*.
