# Supplementary material for: Crystal structures of human ENPP1 in apo and bound forms
Source: Acta Crystallogr D Struct Biol. 2020 Aug 17;76(Pt 9):889–98. doi: 10.1107/S2059798320010505 (PMC7466750; doi:10.1107/S2059798320010505)
Supplement: Supplementary file 1 [file d-76-00889-sup1.pdf]

Acta Cryst D

STRUCTURAL BIOLOGY

Volume 76 (2020)

Supporting information for article:

Crystal structures of human ENPP1 in apo and bound forms

Matthew L. Dennis, Janet Newman, Olan Dolezal, Meghan Hattarki, Regina N. Surjadi, Stewart D. Nuttall, Tam Pham, Tom Nebl, Michelle Camerino, Poh Sim Khoo, Brendon J. Monahan and Thomas S. Peat

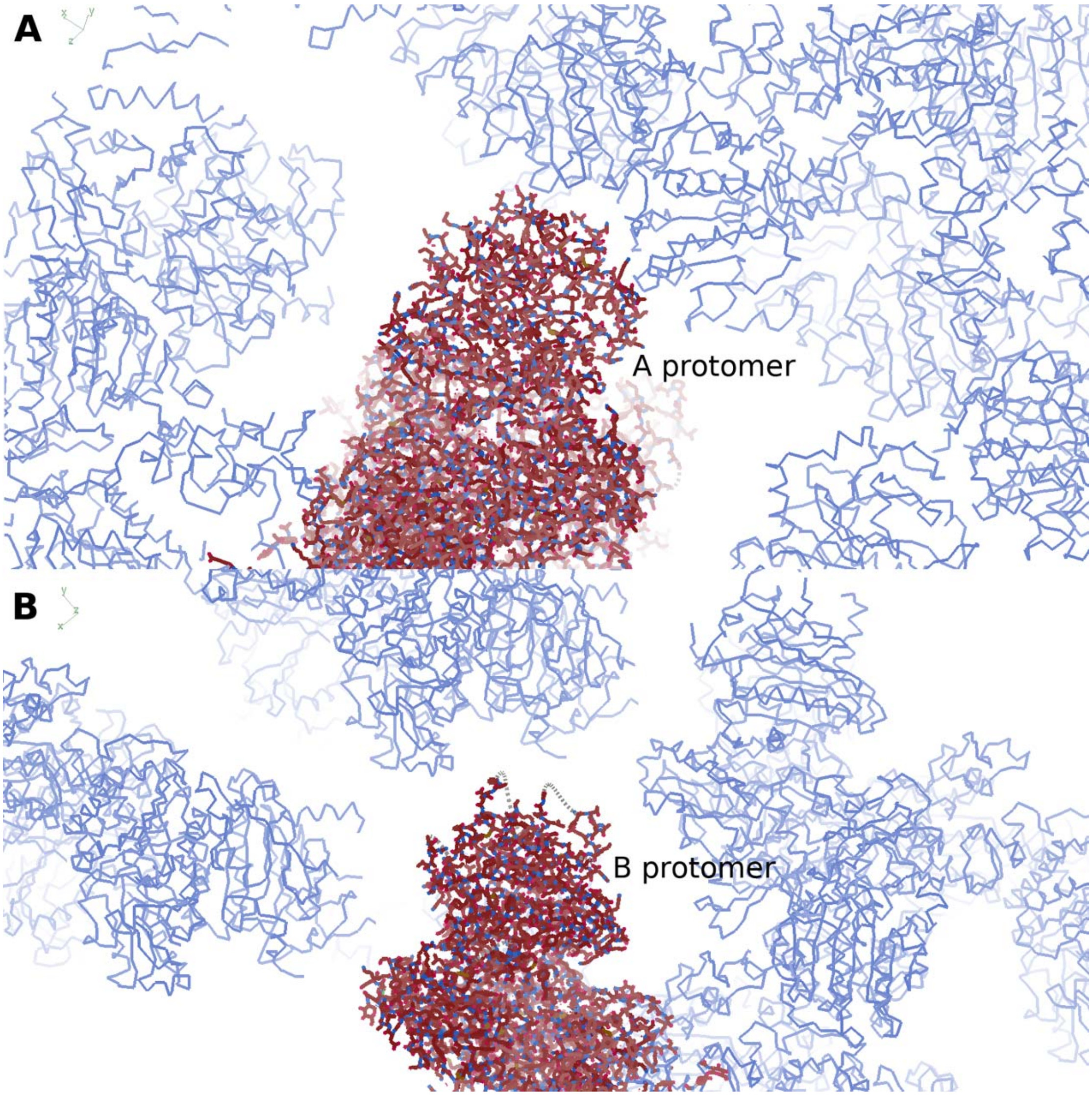

Figure S1 Crystal contacts of hENPP1 (nuclease-like domain). A: Shows the packing of the crystal around the A protomer with the nuclease-like domain in the center of the figure (in red). B: Shows the packing of the crystal around the B protomer with the nuclease-like domain in the center of the figure (in red).

A

Accession	Protein	|Log Prob|	Peptides	% Cov.
P22413	ENPP1$_{98\text{-}925}$	3757	767	96%

```
>sp|P22413|ENPP1_HUMAN Ectonucleotide pyrophosphatase/phosphodiesterase family member

        10         20         30         40         50         60         70         80         90        100
MERDGCAGGGSRGGEGGRAPREGPAGNGRDRGRSHAAEAPGDPQAAASLLAPMDVGEEPLEKAARARTAKDPNTYKVLSLVLSVCVLTTILGCIFGLKPS
       110        120        130        140        150        160        170        180        190        200
CAKEVKSCKGRCFERTFGNCRCDAACVELGNCCLDYQETCIEPEHIWTCNKFRCGEKRLTRSLCACSDDCKDKGDCCINYSSVCQGEKSWVEEPCESINE
       210        220        230        240        250        260        270        280        290        300
PQCPAGFETPPTLLFSLDGFRAEYLHTWGGLLPVISKLKKCGTYTKNMRPVYPTKTFPNHYSIVTGLYPESHGIIDNKMYDPKMNASFSLKSKEKFNPEW
       310        320        330        340        350        360        370        380        390        400
YKGEPIWVTAKYQGLKSGTFFWPGSDVEINGIFPDIYKMYNGSVPFEERILAVLQWLQLPKDERPHFYTLYLEEPDSSGHSYGPVSSEVIKALQRVDGMV
       410        420        430        440        450        460        470        480        490        500
GMLMDGLKELNLHRCLNLILISDHGMEQGSCKKYIYLNKYLGDVKNIKVIYGPAARLRPSDVPDKYYSFNYEGIARNLSCREPNQHFKPYLKHFLPKRLH
       510        520        530        540        550        560        570        580        590        600
FAKSDRIEPLTFYLDPQWQLALNPSERKYCGSGFHGSDNVFSNMQALFVGYGPGFKHGIEADTFENIEVYNLMCDLLNLTPAPNNGTHGSLNHLLKNPVY
       610        620        630        640        650        660        670        680        690        700
TPKHPKEVHPLVQCPFTRNPRDNLGCSCNPSILPIEDFQTQFNLTVAEEKIIKHETLPYGRPRVLQKENTICLLSQHQFMSGYSQDILMPLWTSYTVDRN
       710        720        730        740        750        760        770        780        790        800
DSFSTEDFSNCLYQDFRIPLSPVHKCSFYKNNTKVSYGFLSPPQLNKNSSGIYSEALLTTNIVPMYQSFQVIWRYFHDTLLRKYAEERNGVNVVSGPVFD
       810        820        830        840        850        860        870        880        890        900
FDYDGRCDSLENLRQKRRVIRNQEILIPTHFFIVLTSCKDTSQTPLHCENLDTLAFILPHRTDNSESCVHGKHDSSWVEELLMLHRARITDVEHITGLSF
       910        920
YQQRKEPVSDILKLKTHLPTFSQED
```

B

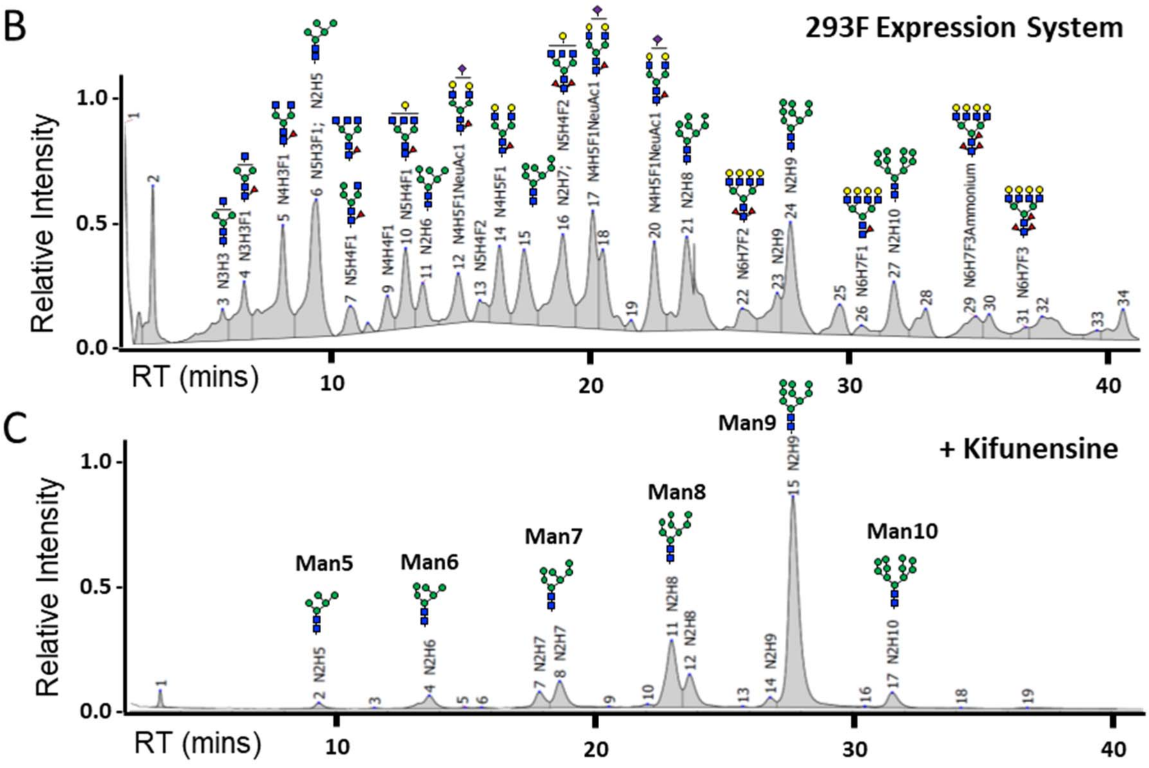

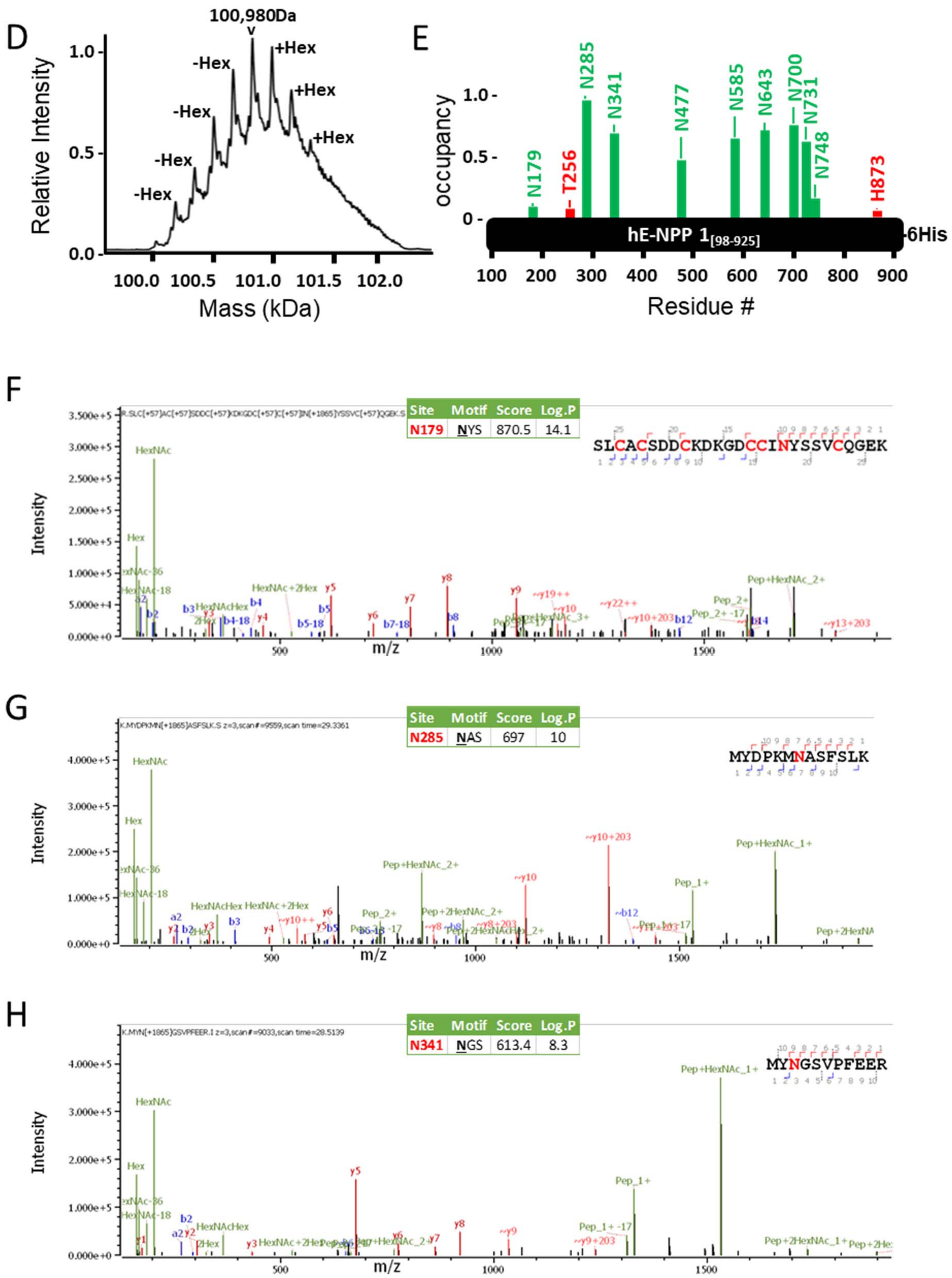
D
100,980Da
Relative Intensity
Mass (kDa)
-Hex
+Hex
E
occupancy
N179
T256
N285
N341
N477
N585
N643
N700
N731
N748
H873
hE-NPP 1[98-925]
6His
Residue #
F
Site Motif Score Log.P
N179 NYS 870.5 14.1
SLCACSDDCKDKGDCCINYSSVCQGEK
Intensity
m/z
G
Site Motif Score Log.P
N285 NAS 697 10
MYDPKMNASFSLK
Intensity
m/z
H
Site Motif Score Log.P
N341 NGS 613.4 8.3
MYNGSVPFEER
Intensity
m/z

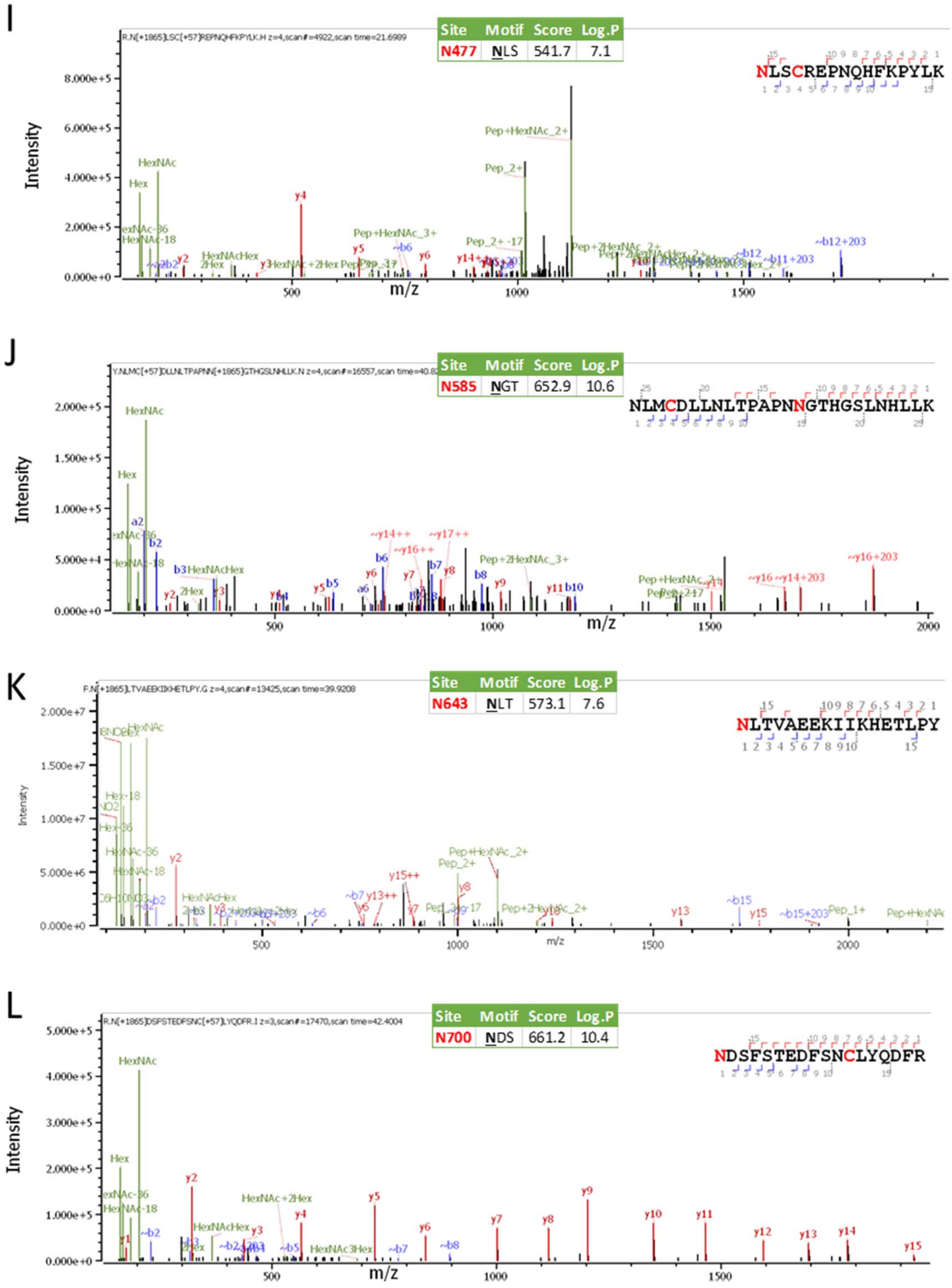
I
R.N[+1865]LSC[+57]REPNQHFKPYLK.H z=4,scan#=4922,scan time=21.6989
Site Motif Score Log.P
N477 NLS 541.7 7.1
NLSCREPNQHFKPYLK
Intensity
m/z
J
Y.NLMC[+57]DLLNLTPAPNN[+1865]GTHGSLNHLLK.N z=4,scan#=16557,scan time=40.8
Site Motif Score Log.P
N585 NGT 652.9 10.6
NLMCDLLNLTPAPNNGTHGSLNHLLK
Intensity
m/z
K
F.N[+1865]LTVAEEKIIKHETLPY.G z=4,scan#=13425,scan time=39.9208
Site Motif Score Log.P
N643 NLT 573.1 7.6
NLTVAEEKIIKHETLPY
Intensity
m/z
L
R.N[+1865]DSFSTEDFSNC[+57]LYQDFR.I z=3,scan#=17470,scan time=42.4004
Site Motif Score Log.P
N700 NDS 661.2 10.4
NDSFSTEDFSNCLYQDFR
Intensity
m/z

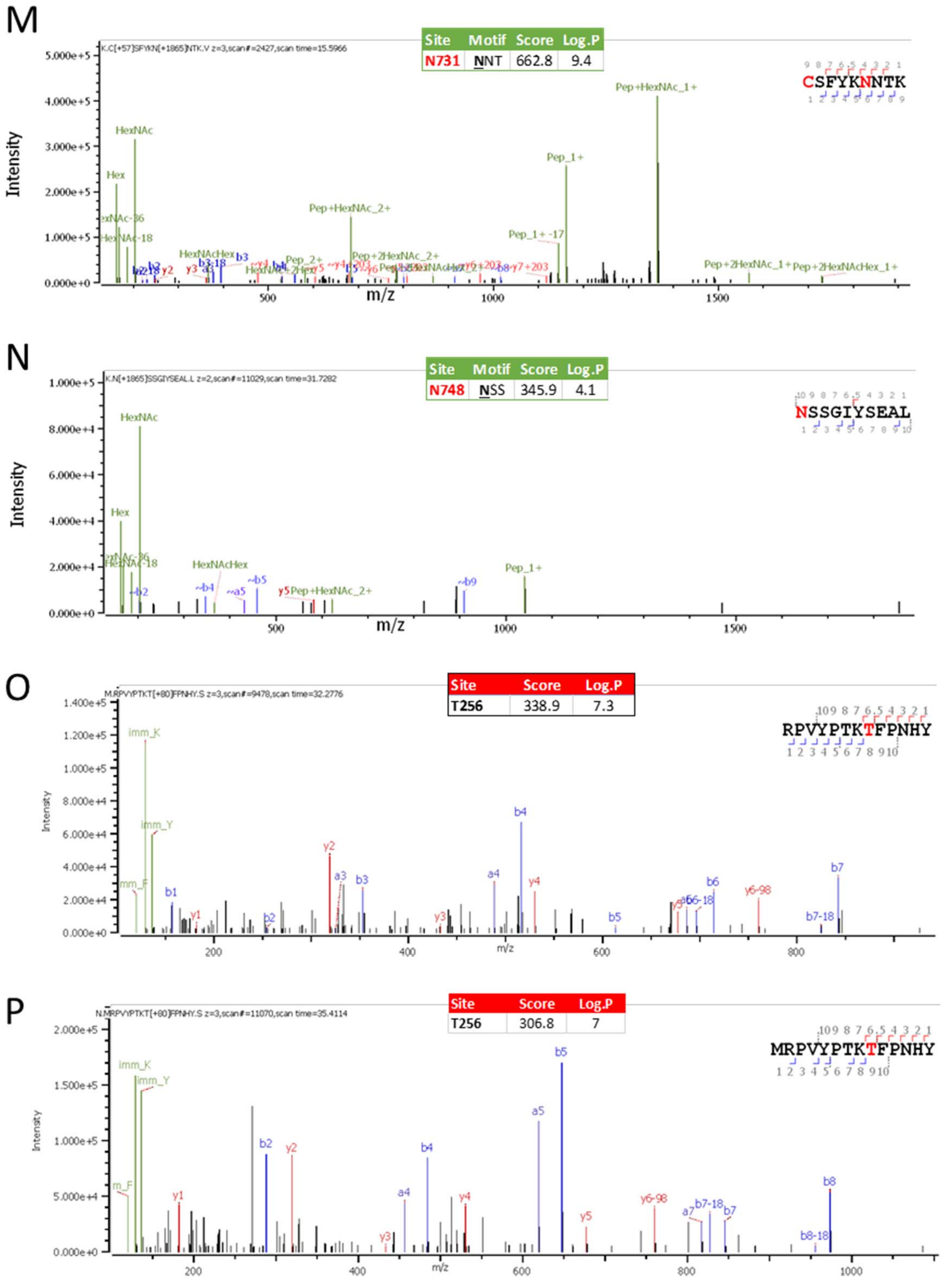
M
K.C[+57]SFYKN[+1865]NTK.V z=3,scan#=2427,scan time=15.5966
Site Motif Score Log.P
N731 NNT 662.8 9.4
CSFYKNNTK
Intensity
m/z
N
K.N[+1865]SSGIYSEAL.L z=2,scan#=11029,scan time=31.7282
Site Motif Score Log.P
N748 NSS 345.9 4.1
NSSGIYSEAL
Intensity
m/z
O
M.RPVYPTKT[+80]FPNHY.S z=3,scan#=9478,scan time=32.2776
Site Score Log.P
T256 338.9 7.3
RPVYPTKTFPNHY
Intensity
m/z
P
N.MRPVYPTKT[+80]FPNHY.S z=3,scan#=11070,scan time=35.4114
Site Score Log.P
T256 306.8 7
MRPVYPTKTFPNHY
Intensity
m/z

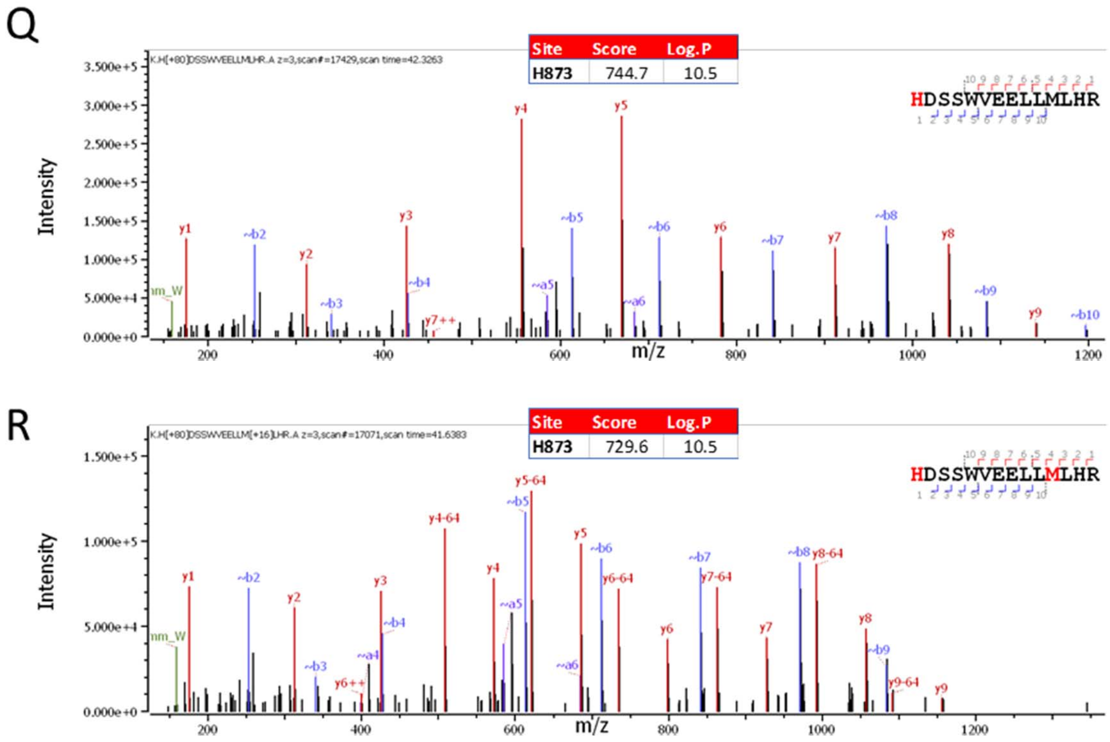

Figure S2 Mass spectrometric analysis. **A:** Peptide sequencing of a kifunensine-treated, affinity purified hENPP1-HIS preparation by LC-MSMS confidently identified 767 unique peptides with 96% sequence coverage matching of hENPP1 residues 98-925 (green). Nine predicted N-linked glycosylation sites of hENPP1 are highlighted in yellow, based on UniProt entry P22413. **B:** N-linked glycoprofiling of hENPP1 expressed in Freestyle 293 cells identified over 30 different glycan structures, including predominantly high-mannose and complex, core-fucosylated glycan structures. **C:** Kifunensine treatment significantly reduced N-glycan heterogeneity and resulted in hENPP1 decorated with high-mannose-type glycan structures of Man9 > Man8 > Man7 type. **D:** Intact mass determination of a kifunensine-treated hENPP1 154-925 construct is consistent with 13.8-15.0kDa N-linked glycan mass additions (+/- Hex). The major observed mass of 100,980Da matches the expected protein mass (MW~86,556.5Da) decorated with 4xMan8 + 4xMan9 N-linked glycans. **E:** A diagram depicting the relative occupancy of N-linked glycosylation sites (green) or phosphorylation sites (red) of hENPP1 based on spectrum counting (e.g. fraction of modified versus total peptide spectra matches per site; height of bars and whiskers correspond to average +/- standard error of three independent biological replicates). **F-N:** Highly confident LC-MSMS evidence spectra showing Man9-modified glycopeptide sequences for glycosylation sites N179, N285, N341, N477, N585, N700, N731 and N48. **O-R:** LC-MSMS evidence for peptide sequences containing phosphorylated T256 or H873.

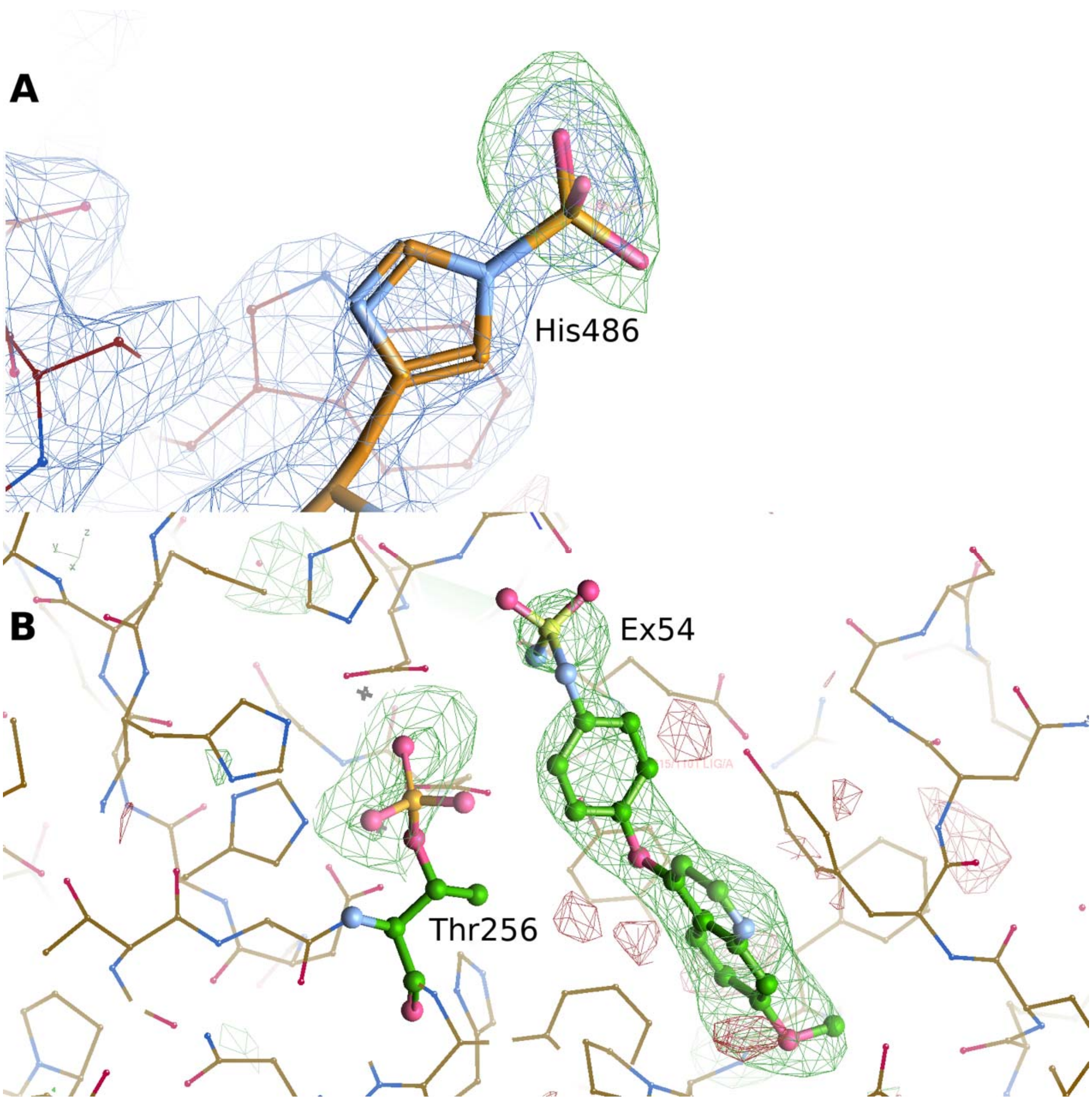

Figure S3 Difference density for phosphorylation sites A: Difference density map (green) set at 3 σ for His486 showing significant difference density in the original MR map generated (difference density for the phosphate is still seen at 7 σ). The blue density is set at 1.5 σ. B: Difference density map set at 3 σ for both Thr256 and Ex54 in the catalytic site of hENPP1. Figure was generated using Coot and The Gimp.

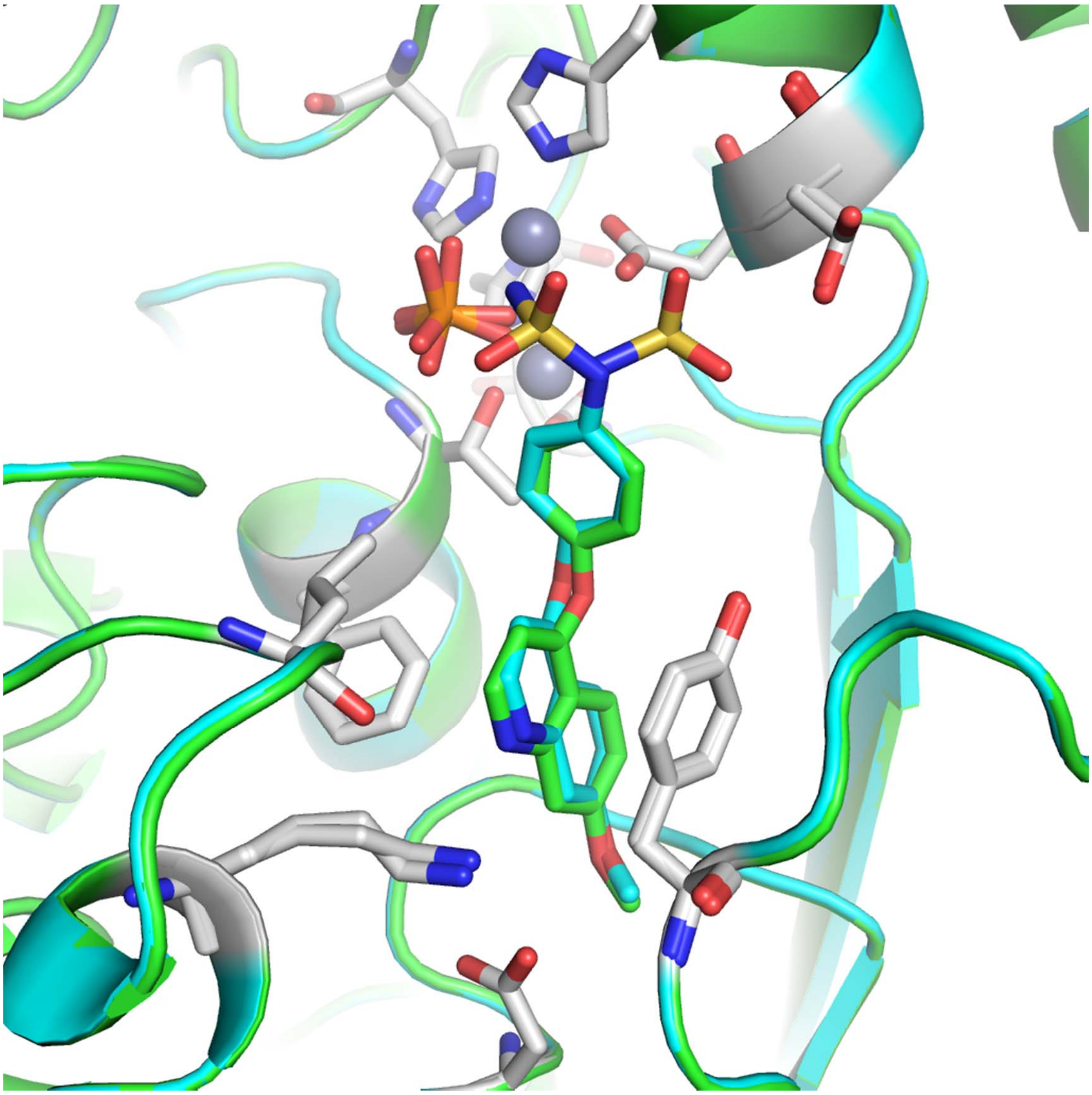

Figure S4 Ex54 sulfamide movement. Variable sulfamide position of Ex54. The A and B protomers were superposed using the SSM algorithm in Coot. There is little variation between these active sites, but one can see that the sulfamide position of the two Ex54 molecules is quite different. Figure was generated with PyMol.

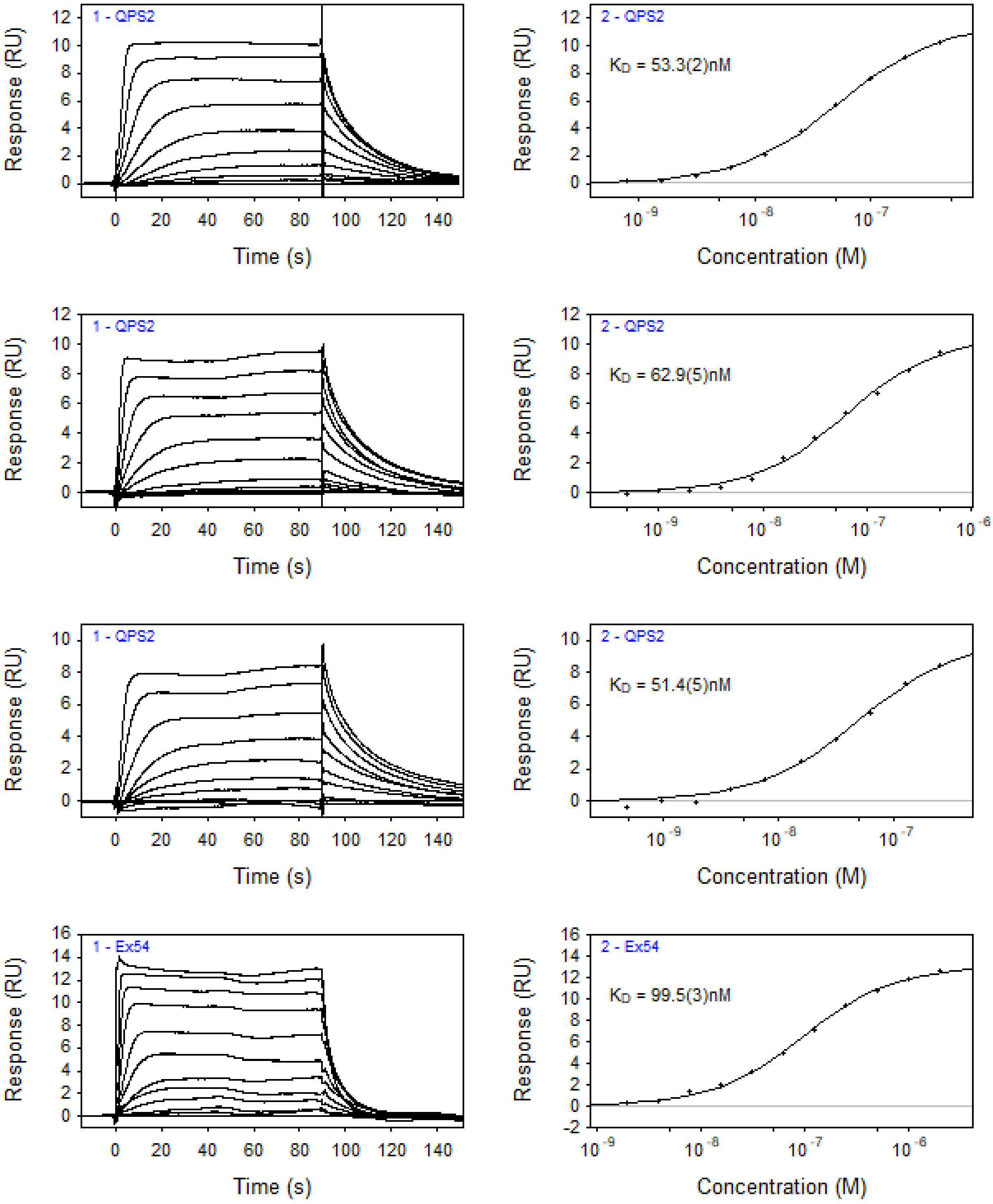
1 - QPS2
2 - QPS2
KD = 53.3(2)nM
1 - QPS2
2 - QPS2
KD = 62.9(5)nM
1 - QPS2
2 - QPS2
KD = 51.4(5)nM
1 - Ex54
2 - Ex54
KD = 99.5(3)nM
Response (RU)
Time (s)
Concentration (M)

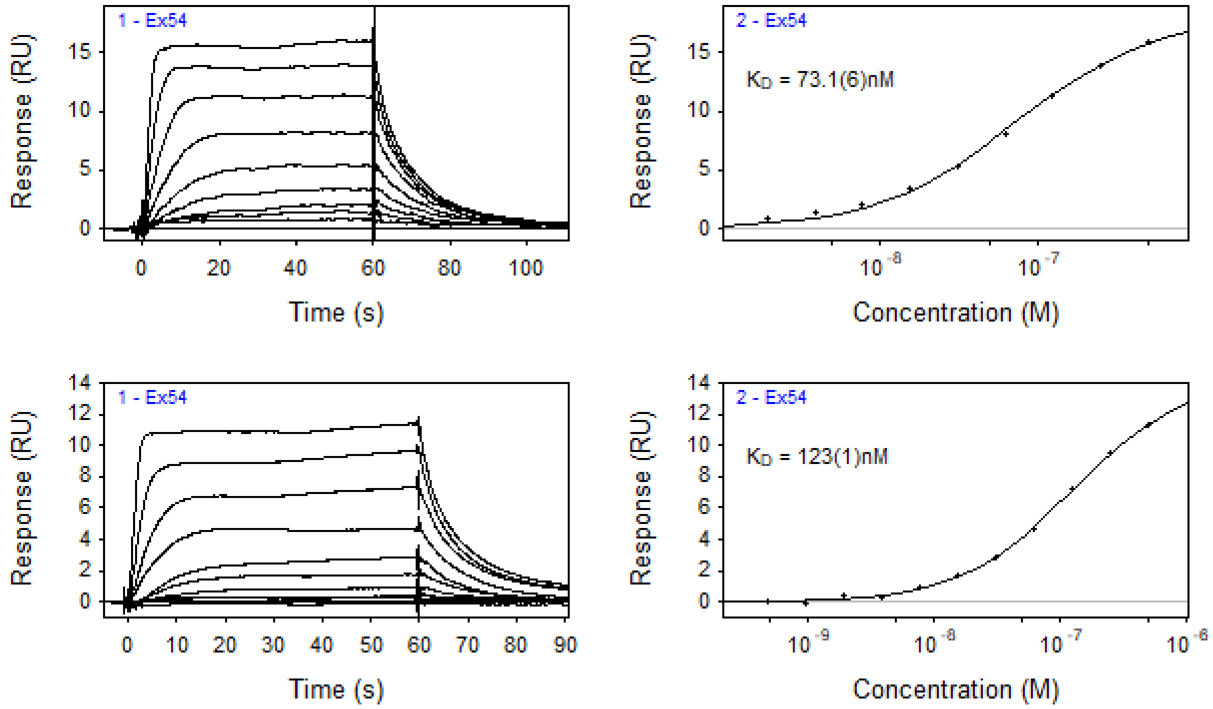
1 - Ex54
Response (RU)
Time (s)
2 - Ex54
K_D = 73.1(6)nM
Response (RU)
Concentration (M)
1 - Ex54
Response (RU)
Time (s)
2 - Ex54
K_D = 123(1)nM
Response (RU)
Concentration (M)

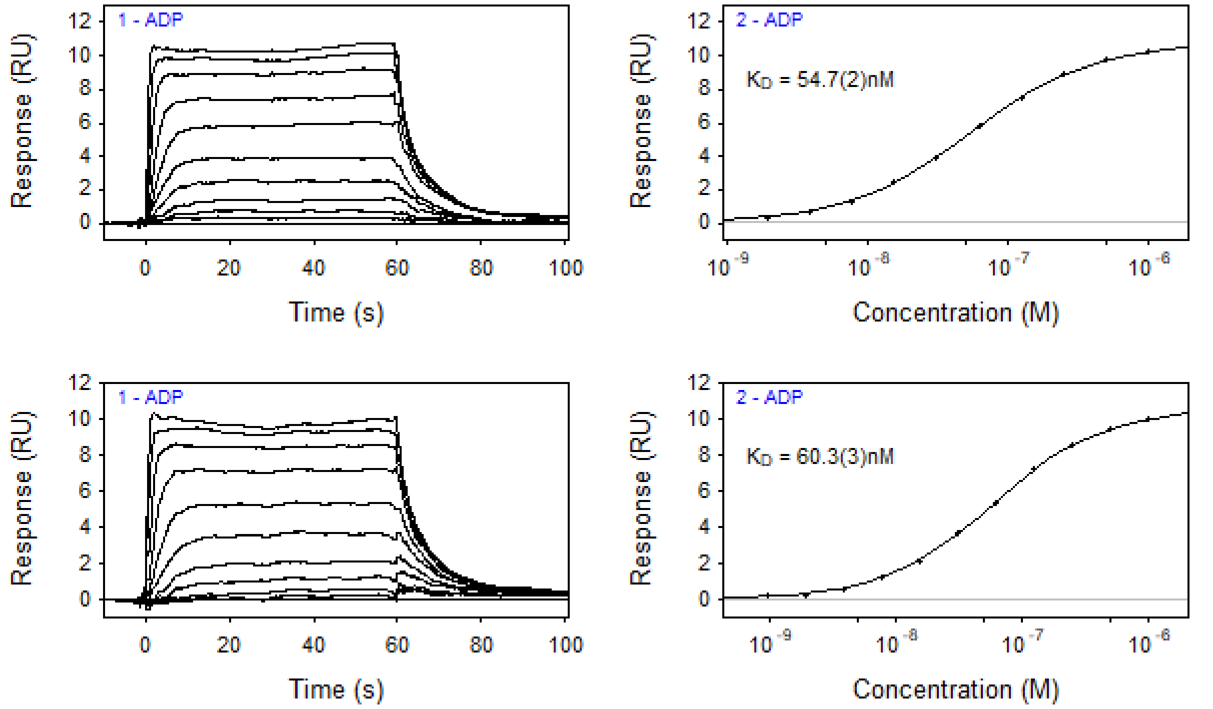
1 - ADP
Response (RU)
Time (s)
2 - ADP
K_D = 54.7(2)nM
Response (RU)
Concentration (M)
1 - ADP
Response (RU)
Time (s)
2 - ADP
K_D = 60.3(3)nM
Response (RU)
Concentration (M)

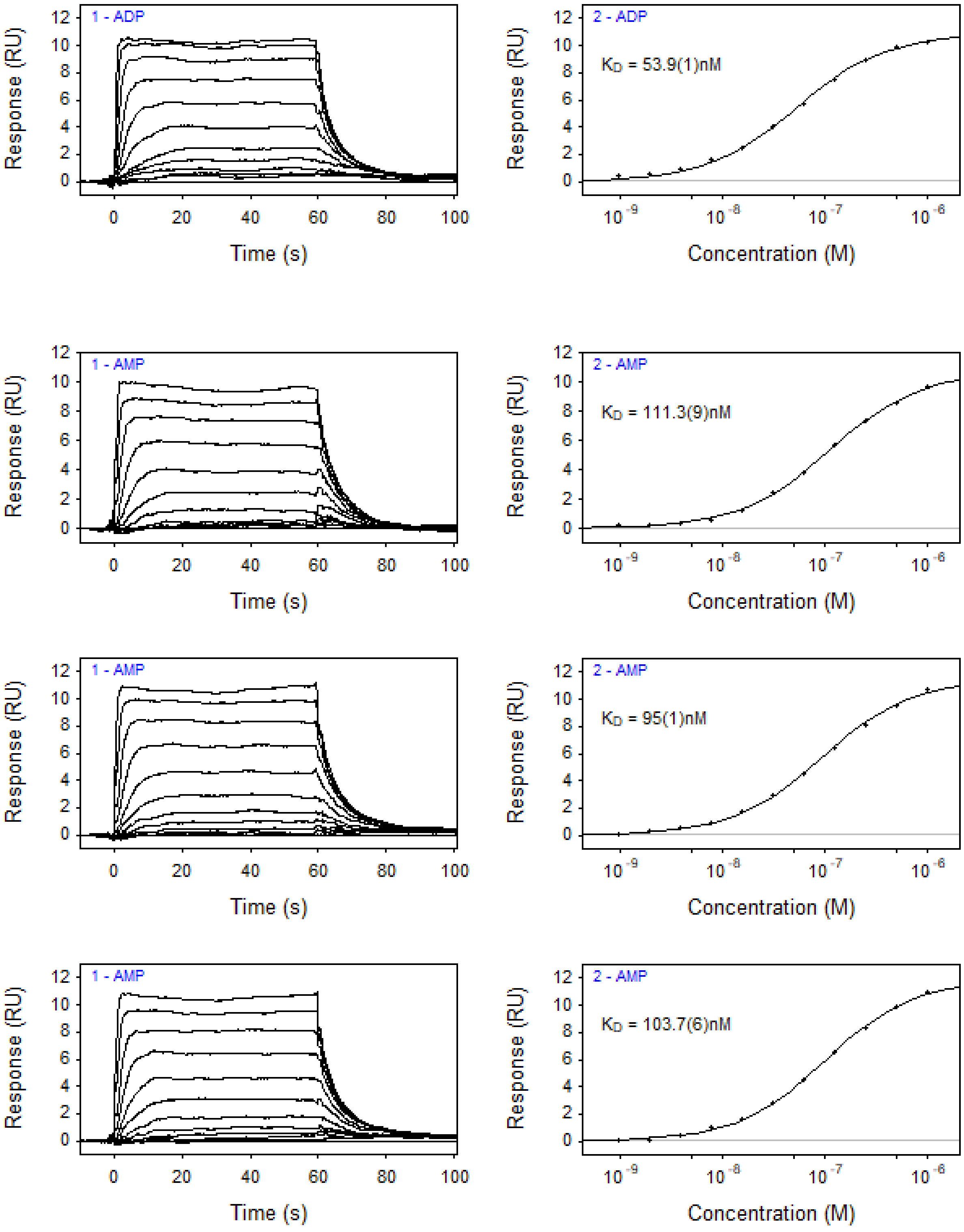
1 - ADP
Response (RU)
Time (s)
2 - ADP
KD = 53.9(1)nM
Concentration (M)
1 - AMP
2 - AMP
KD = 111.3(9)nM
1 - AMP
2 - AMP
KD = 95(1)nM
1 - AMP
2 - AMP
KD = 103.7(6)nM

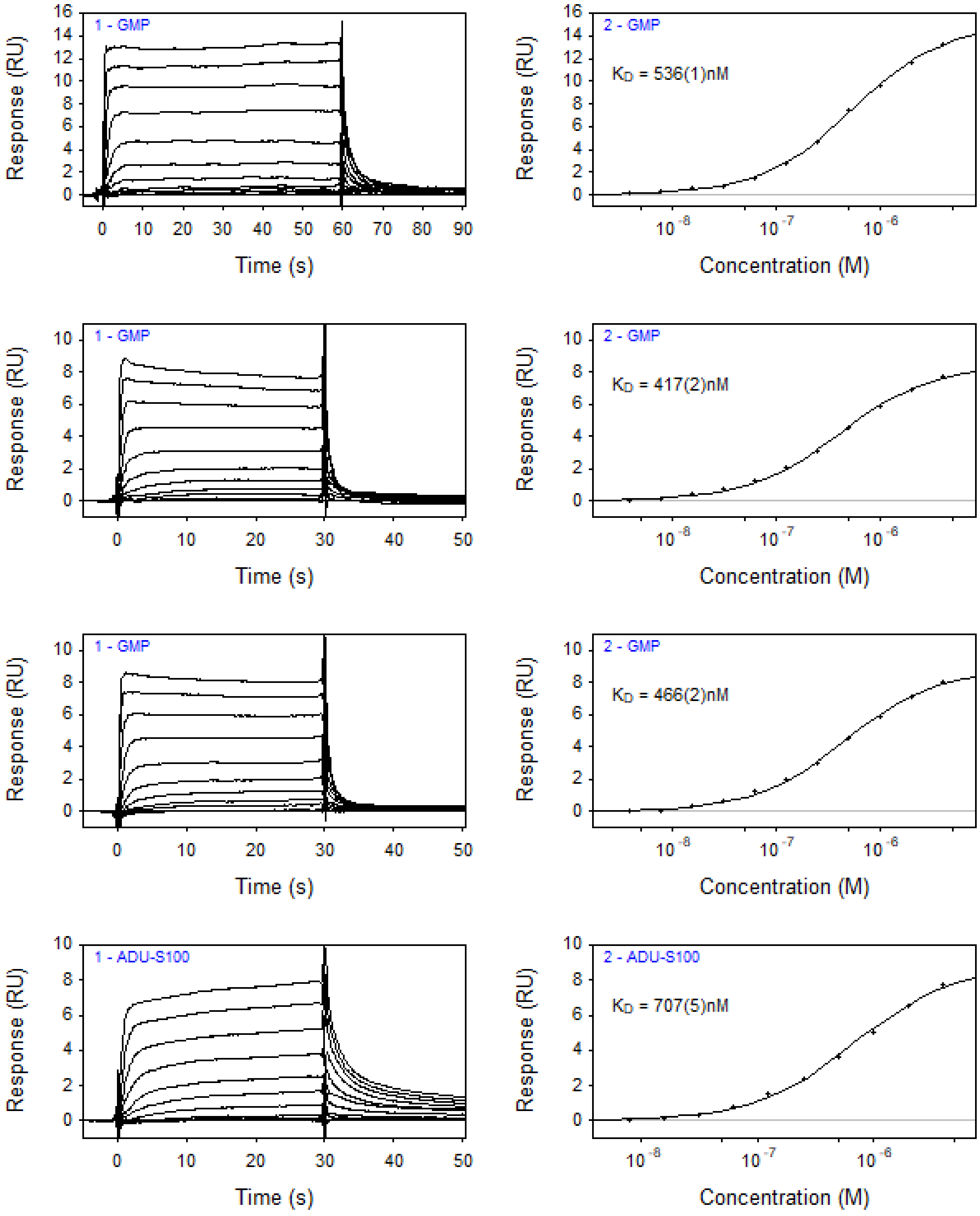
1 - GMP
Response (RU)
Time (s)
2 - GMP
K_D = 536(1)nM
Concentration (M)
1 - GMP
Response (RU)
Time (s)
2 - GMP
K_D = 417(2)nM
Concentration (M)
1 - GMP
Response (RU)
Time (s)
2 - GMP
K_D = 466(2)nM
Concentration (M)
1 - ADU-S100
Response (RU)
Time (s)
2 - ADU-S100
K_D = 707(5)nM
Concentration (M)

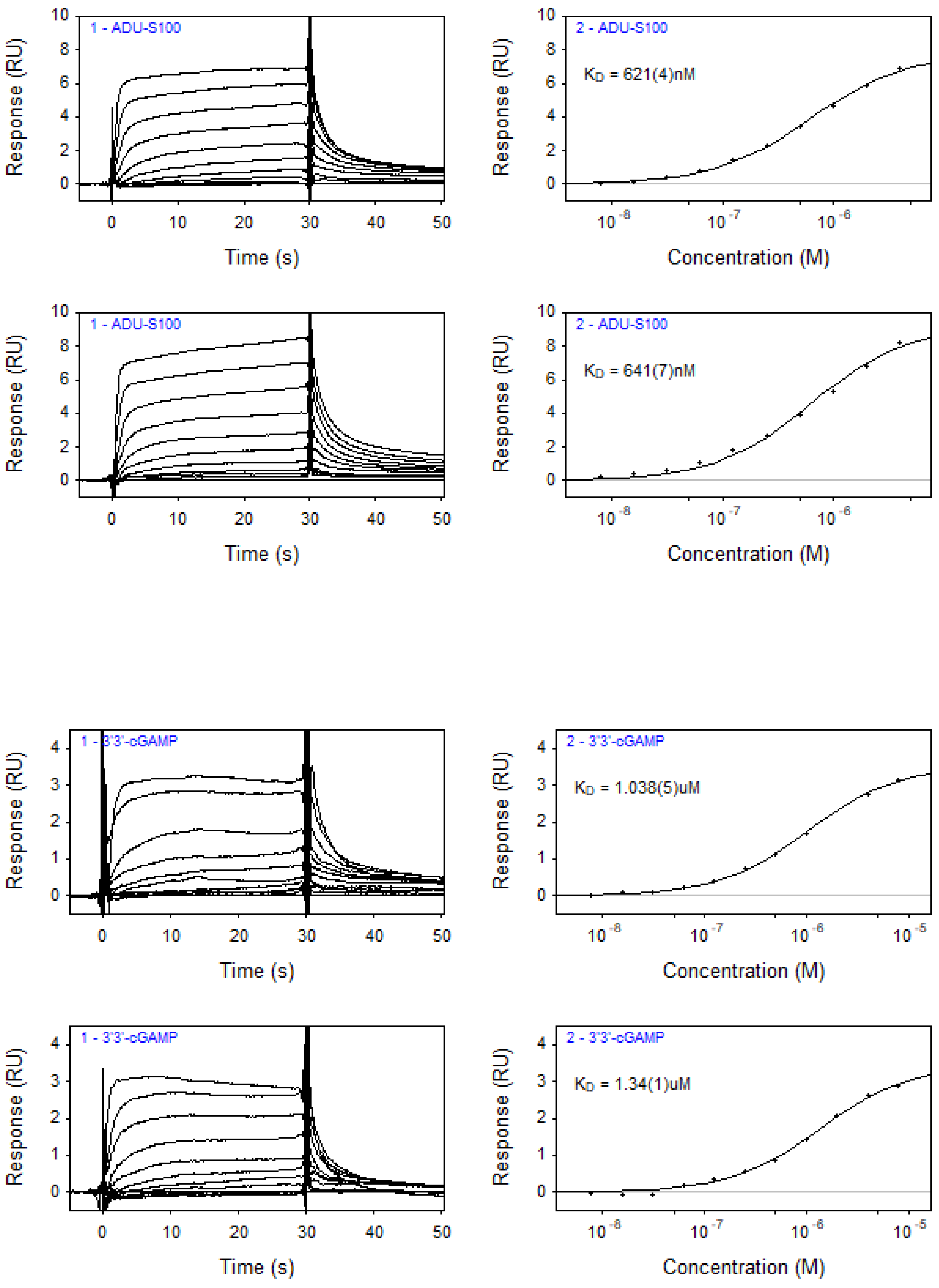
1 - ADU-S100
Response (RU)
Time (s)
2 - ADU-S100
KD = 621(4)nM
Concentration (M)
1 - ADU-S100
Response (RU)
Time (s)
2 - ADU-S100
KD = 641(7)nM
Concentration (M)
1 - 3'3'-cGAMP
Response (RU)
Time (s)
2 - 3'3'-cGAMP
KD = 1.038(5)uM
Concentration (M)
1 - 3'3'-cGAMP
Response (RU)
Time (s)
2 - 3'3'-cGAMP
KD = 1.34(1)uM
Concentration (M)

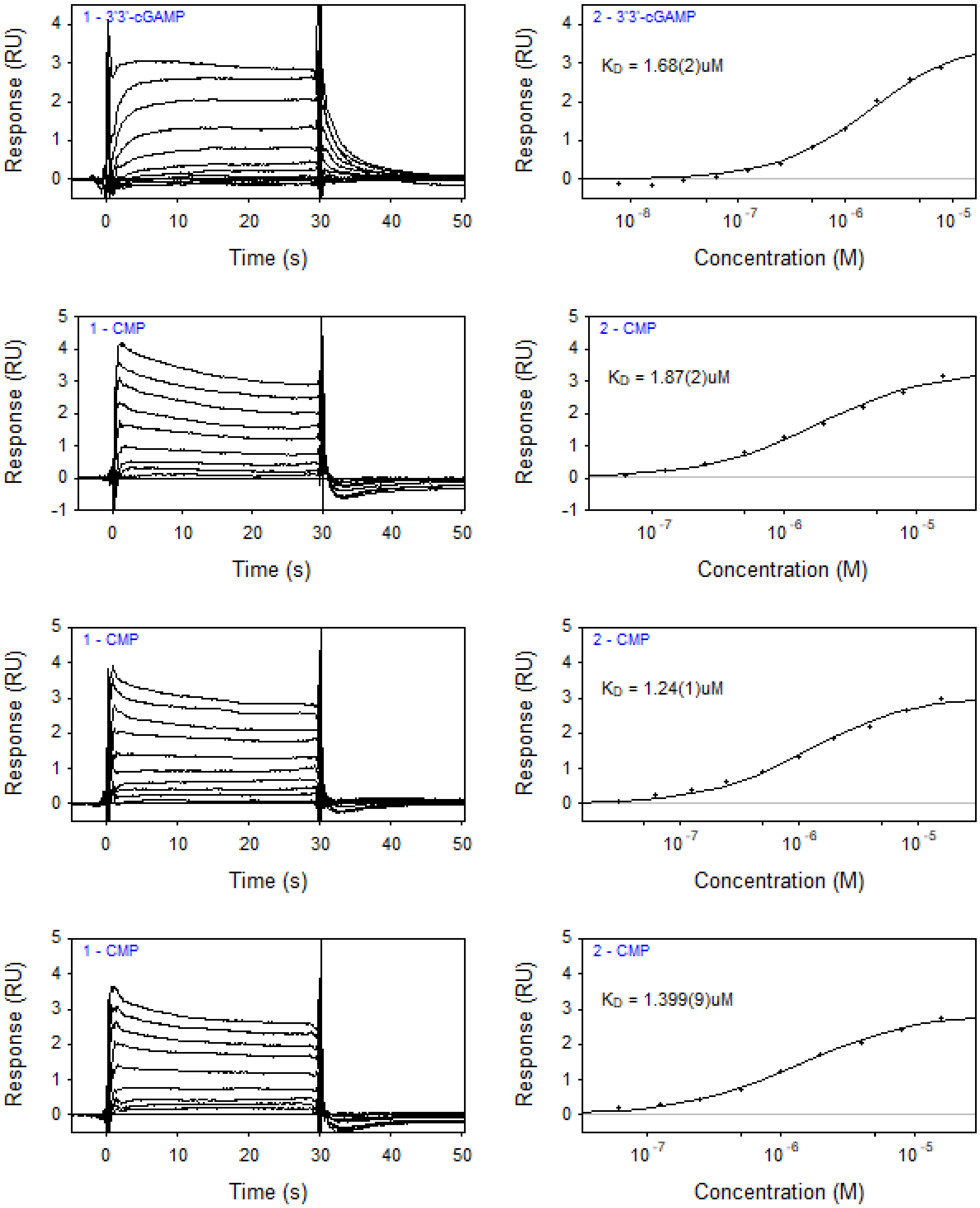
1 - 3'3'-cGAMP
Response (RU)
Time (s)
2 - 3'3'-cGAMP
KD = 1.68(2)uM
Concentration (M)
1 - CMP
2 - CMP
KD = 1.87(2)uM
1 - CMP
2 - CMP
KD = 1.24(1)uM
1 - CMP
2 - CMP
KD = 1.399(9)uM

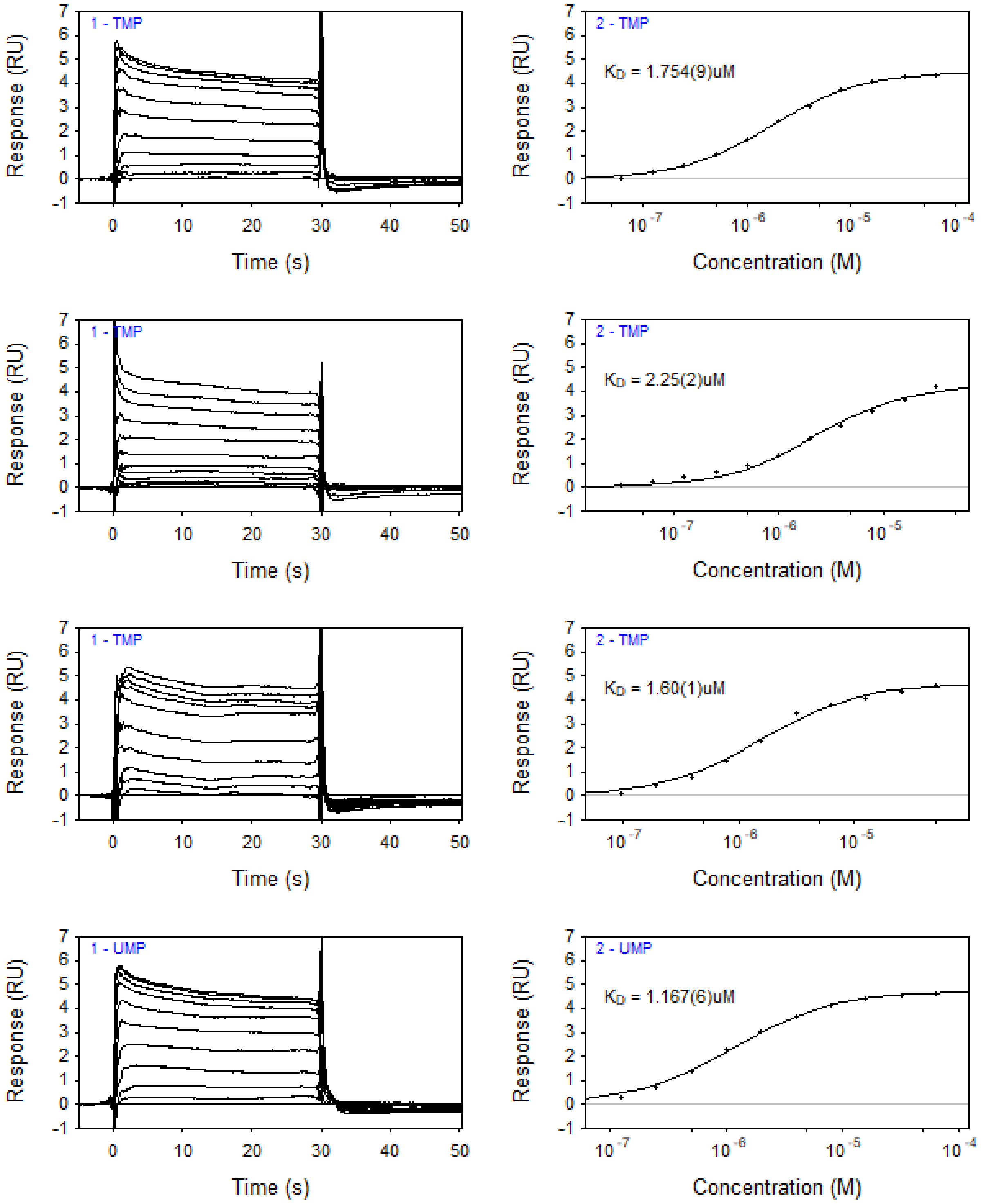
1 - TMP
Response (RU)
Time (s)
2 - TMP
K_D = 1.754(9)uM
Response (RU)
Concentration (M)
1 - TMP
Response (RU)
Time (s)
2 - TMP
K_D = 2.25(2)uM
Response (RU)
Concentration (M)
1 - TMP
Response (RU)
Time (s)
2 - TMP
K_D = 1.60(1)uM
Response (RU)
Concentration (M)
1 - UMP
Response (RU)
Time (s)
2 - UMP
K_D = 1.167(6)uM
Response (RU)
Concentration (M)

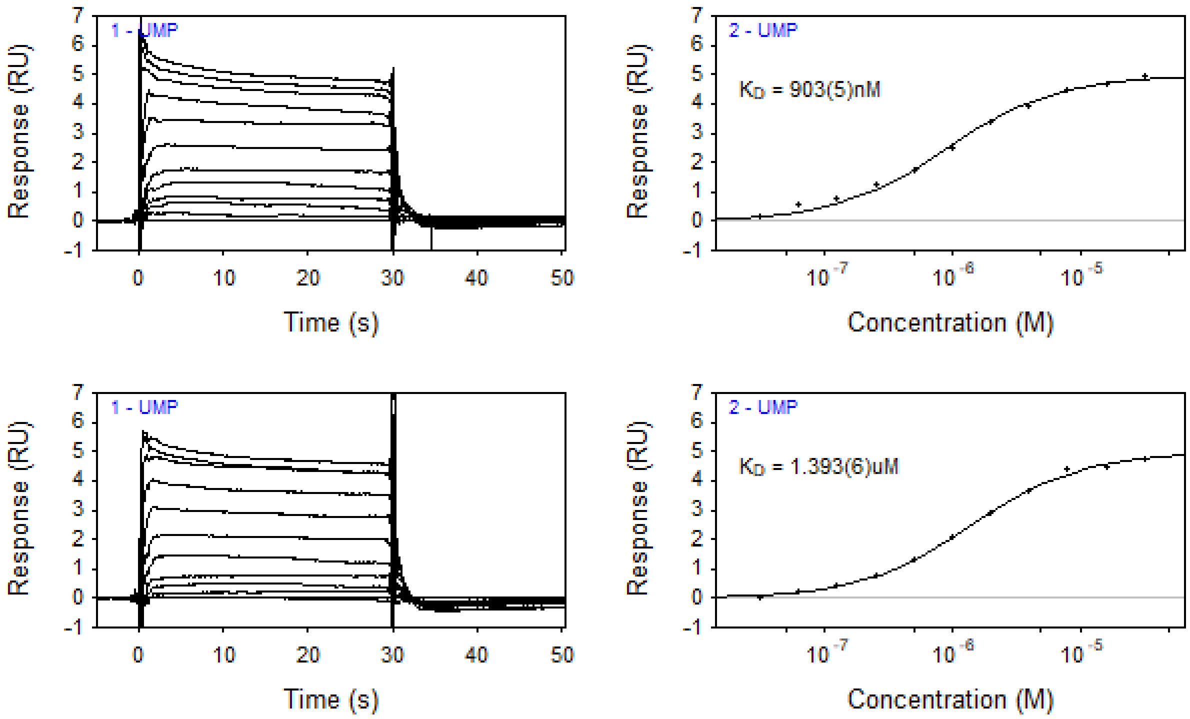

Figure S5 SPR sensorgrams and binding isotherms.

DMSO(0%)

Tm = 59.3(+/-0.08)

DMSO(1%),

Tm = 58.54(+/-0.02)

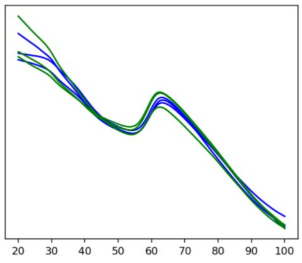

GMP

Tm = 60.34 (+/-0.03)

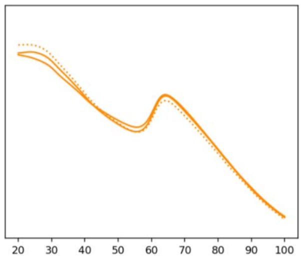

ADU-S100

Tm = 65.57 (+/-0.06)

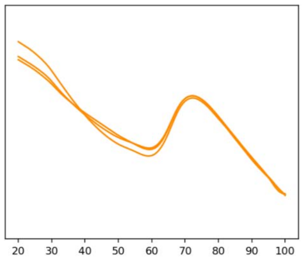

CMP

Tm = 61.38 (+/-0.0)

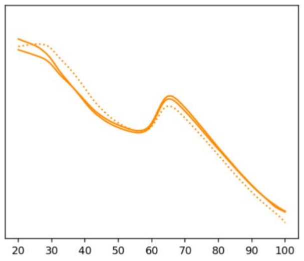

3'3'-cGAMP

Tm = 60.55 (+/-0.02)

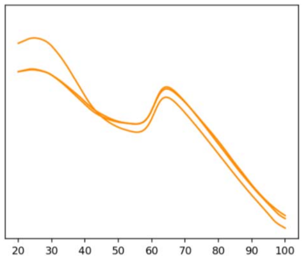

ADP

Tm = 62.6 (+/-0.08)

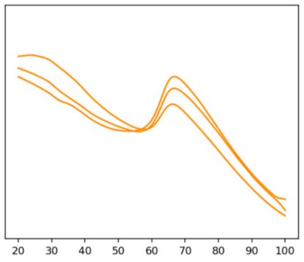

AMP

Tm = 62.01 (+/-0.1)

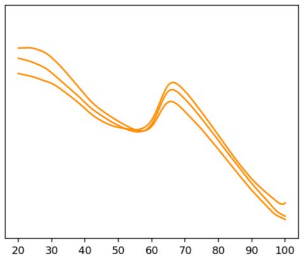

TMP

Tm = 60.54 (+/-0.1)

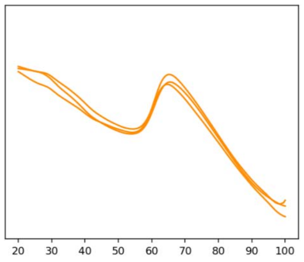

UMP

Tm = 61.05 (+/-0.05)

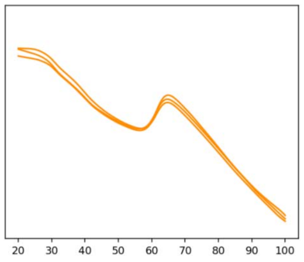

QPS2

Tm = 63.03 (+/-0.13)

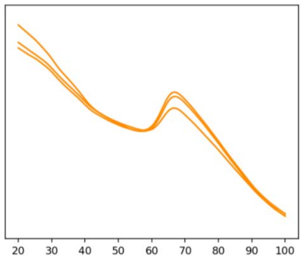

DMSO(1%)

Tm = 58.37(+/-0.07)

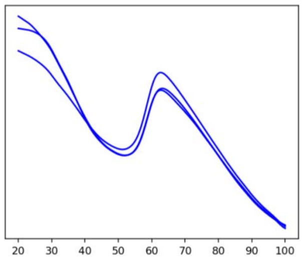

Ex54

Tm = 62.15 (+/-0.03)

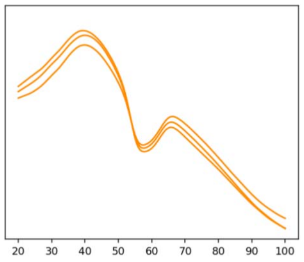

Figure S6 DSF melt curves. The x-axis presents temperature (ºC) and the y-axis presents normalised RFU (relative fluorescence units).
